# Comparison of design approaches for low-cost sampling mechanisms in open-source chemical instrumentation

**DOI:** 10.1016/j.ohx.2021.e00220

**Published:** 2021-08-10

**Authors:** Greggory Murray, Samuel Bednarski, Michael Hall, Samuel W. Foster, SiJun Jin, Joshua J. Davis, Wei Xue, Eric Constans, James P. Grinias

**Affiliations:** a Department of Mechanical Engineering, Rowan University, Glassboro, NJ, United States; b Department of Mechanical Engineering, Rose-Hulman Institute of Technology, Terre Haute, IN, United States; c Department of Chemistry & Biochemistry, Rowan University, Glassboro, NJ, United States

**Keywords:** Autosampler, Droplet microfluidics, Robotics, Chemical analysis

## Abstract

Robotic positioning systems are used in a variety of chemical instruments, primarily for liquid handling purposes, such as autosamplers from vials or well plates. Here, two approaches to the design of open-source autosampler positioning systems for use with 96-well plates are described and compared. The first system, a 3-axis design similar to many low-cost 3D printers that are available on the market, is constructed using an aluminum frame and stepper motors. The other system relies upon a series of 3D printed parts to achieve movement with a series of linker arms based on Selective Compliance Assembly Robot Arm (SCARA) design principles. Full printer design files, assembly instructions, software, and user directions are included for both samplers. The positioning precision of the 3-axis system is better than the SCARA mechanism due to finer motor control, albeit with a slightly higher cost of materials. Based on the improved precision of this approach, the 3-axis autosampler system was used to demonstrate the generation of a segmented flow droplet stream from adjacent wells within a 96-well plate.

## Graphical abstract

**Figure:**
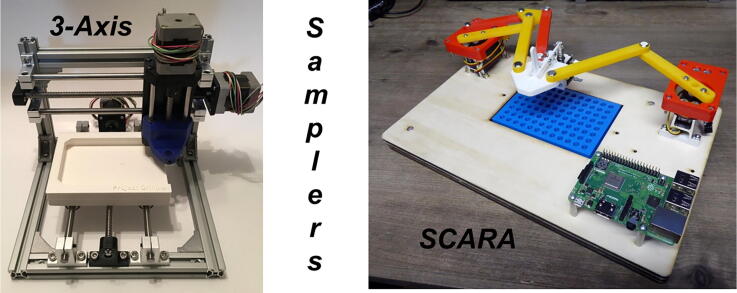

## Specifications table

**Table:** 

Hardware Name	• 3-Axis Autosampler • SCARA Autosampler
Subject Area	• Chemistry and Biochemistry • Educational Tools and Open-Source Alternatives to Existing Infrastructure
Hardware Type	• Chemical sample handling and preparation
Open Source License	• *CC BY 4.0*
Cost of Hardware	• 3-Axis Autosampler: ∼$335 • SCARA Autosampler: ∼$300
Source File Repository	*https://doi.org/10.17632/vfn2g8xg36.1*

## 1 Hardware in context

Many modern chemical instruments include the use of autosamplers to introduce samples for analysis [1], including gas chromatographs (GCs), liquid chromatographs (LCs), mass spectrometers (MSs), capillary electrophoresis (CE) instruments, and flow injection analyzers (FIAs). The movement of these samplers typically relies on either a 3-axis linear motion system or a 2-axis linear motion system with a third angular rotation mechanism, both with the goal of sampling specific positions in sample trays or well plates [1]. Fraction collectors, in which the eluent from a chromatographic column or other fluidic stream is collected into separate tubes or wells over time, operate under similar principles. With the advent of 3D printing, the cost and complexity of these types of motion systems (and their associated motors) has dropped dramatically, providing an opportunity to develop open-source solutions for autosampling [2], [3], [4], [5], [6], liquid sample manipulation [3], [7], [8], and fraction collection [4], [5]. Additional reports of 3-axis motion systems based on 3D printers that have been adapted for chemical research include mass spectrometry sampling [9], [10], matrix deposition for matrix-assisted laser desorption ionization (MALDI) [11], chromatographic fraction collection [12], sample preparation and injection [13], applications in thin-layer chromatography (TLC) [14], [15], and nucleic acid sample processing [16], [17]. Open-source chemistry applications of angular rotation mechanisms have mainly been demonstrated through the use of sampling robotic arms thus far [18], [19]. From these various reports, it is clear that the application of these approaches in chemical research are widespread and will continue to grow with the advent of open-source chemical instrumentation [20]. A key driving factor in the open-source hardware movement is a reduction in cost for laboratory tools. However, these open-source, low-cost options must still perform at acceptable levels to adequately complete desired tasks. In this report, the designs for an open-source 3-axis motion system using stepper motors similar to those adapted from 3D printers and a system using an angular rotation mechanism based on a parallel Selective Compliance Assembly Robot Arm (SCARA) mechanism [21] controlled with servomotors are both described. The systems are compared in terms of movement precision, and the 3-axis system is also demonstrated for potential use in segmented flow microfluidic workflows.

## 2 Hardware description

**Fig. 1:**
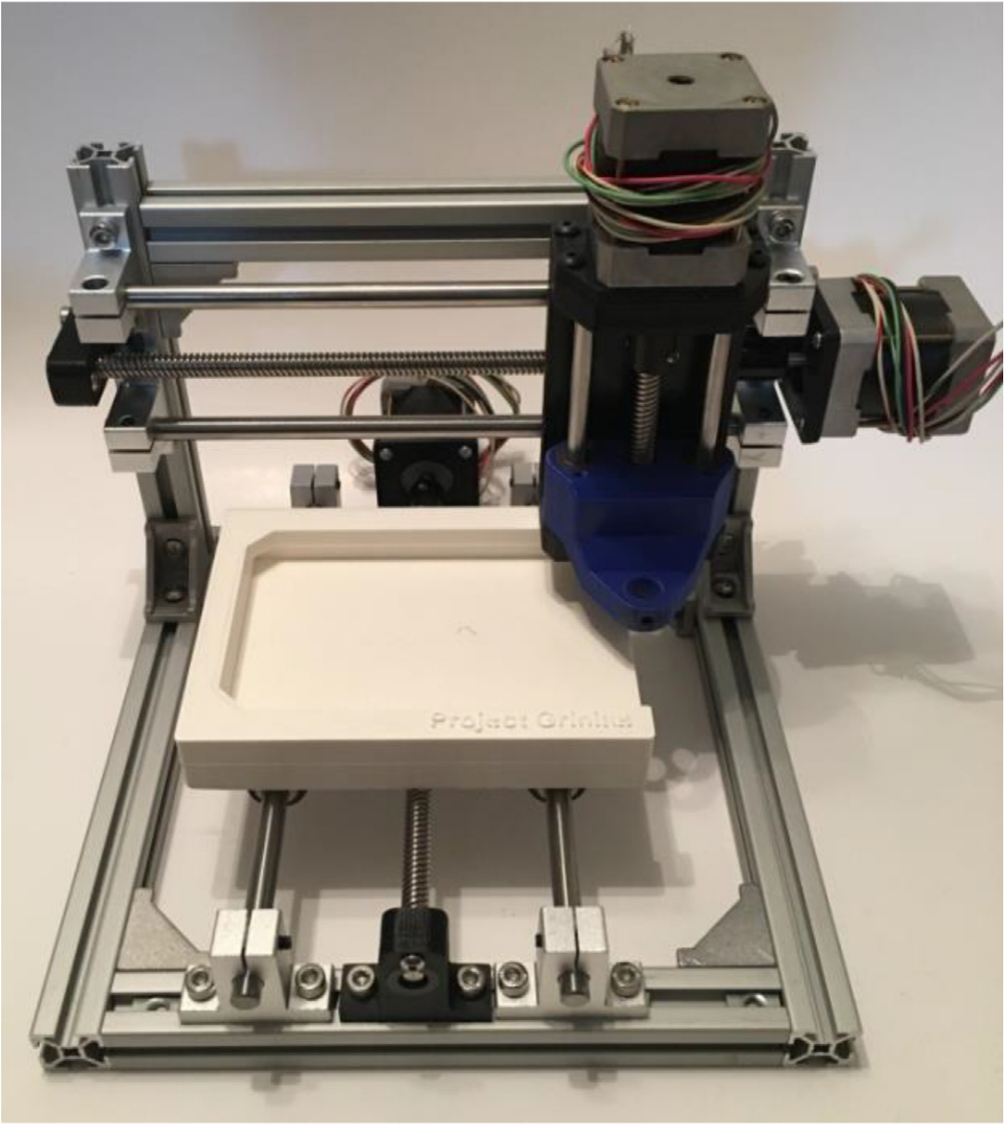
Completed 3-axis autosampler system.

**Fig. 2:**
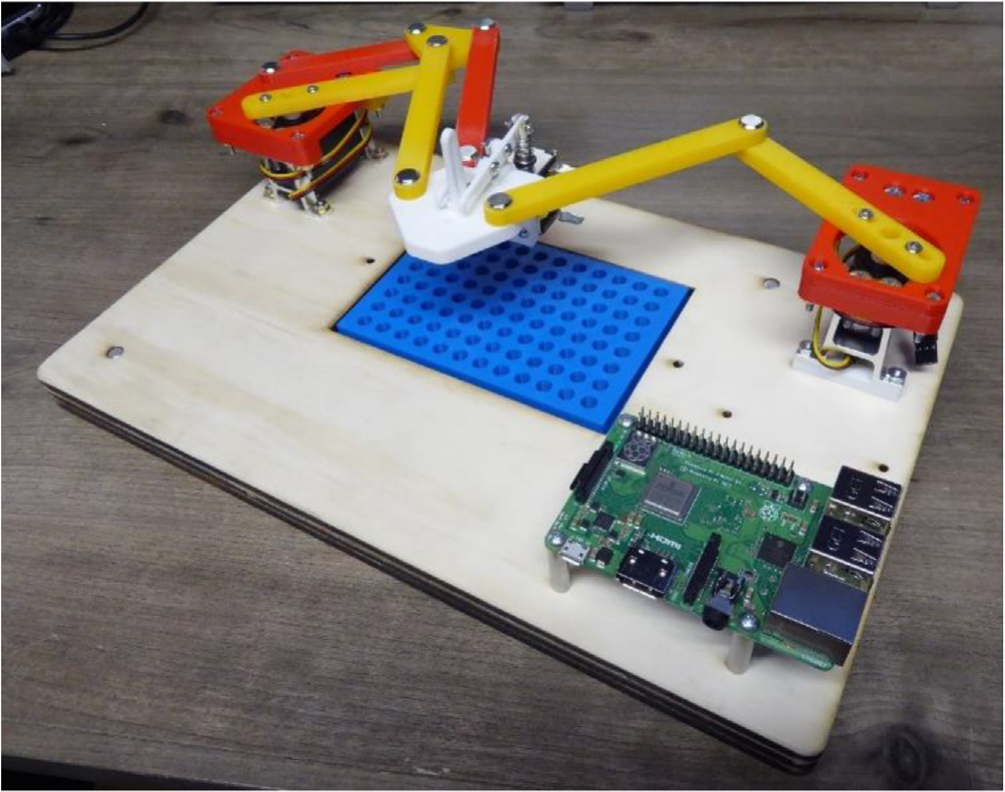
Completed SCARA autosampler system.

The 3-axis autosampler design (Fig. 1) was ctesian plane movement system using stepper motors that is common in many commercially available 3D printers. The SCARA design (Fig. 2) relies upon angular rotation movement between multiple linker arms for x-axis and y-axis servomotor positioning, with two-position movement in the z-axis controlled by a solenoid. Both systems cost significantly less than commercial autosampler systems and are comparable in price to approaches that rely upon the modification of commercial low-cost 3D printers. With the foundation provided for each approach here, they can be further modified to accommodate additional functionality, including the many purposes described in ***Section 1***.

The movement precision of these two design approaches has not directly been compared to date in the context of open-source chemical instrumentation. To identify which system provided finer movement control, a process adapted from ISO 9283:1998 [22] was used to determine the movement precision for each approach. As a demonstration of a specific relevant application in the field of microfluidics, the 3-axis system was applied toward the generation of segmented flow droplet streams from a 96-well plate, an approach with implications in high-throughput screening (HTS) [23], [24], [25], [26], [27], [28], [29].

## 3 Design files

The files listed in Table 1 are used in the construction of the 3-axis autosampler system described in Sections 5.1.1-5.1.4.

**Table 1:** Design files for construction of 3-axis autosampler.

Design file name	File type	Open source license	Location of the file
capillary_elec_insert *2 Needed	*STL, SLDPRT*	*CC BY 4.0*	*https://doi.org/10.17632/vfn2g8xg36.1*
*leadscrew_nut_housing_x_axis*	*STL, SLDPRT*	*CC BY 4.0*	*https://doi.org/10.17632/vfn2g8xg36.1*
*leadscrew_nut_housing_y_axis*	*STL, SLDPRT*	*CC BY 4.0*	*https://doi.org/10.17632/vfn2g8xg36.1*
nema_17_xy_mount *2 Needed	*STL, SLDPRT*	*CC BY 4.0*	*https://doi.org/10.17632/vfn2g8xg36.1*
*nema_17_z_mount*	*STL, SLDPRT*	*CC BY 4.0*	*https://doi.org/10.17632/vfn2g8xg36.1*
*process_interface_carriage*	*STL, SLDPRT*	*CC BY 4.0*	*https://doi.org/10.17632/vfn2g8xg36.1*
*well_plate_holder*	*STL, SLDPRT*	*CC BY 4.0*	*https://doi.org/10.17632/vfn2g8xg36.1*
*x_axis_leadscrew_support*	*STL, SLDPRT*	*CC BY 4.0*	*https://doi.org/10.17632/vfn2g8xg36.1*
*x_carriage_frame*	*STL, SLDPRT*	*CC BY 4.0*	*https://doi.org/10.17632/vfn2g8xg36.1*
*y_axis_leadscrew_support*	*STL, SLDPRT*	*CC BY 4.0*	*https://doi.org/10.17632/vfn2g8xg36.1*

The file listed in Table 2 is used to install the software for the 3-axis autosampler system, as described in *Section 5.1.5*.

**Table 2:** Software file for operation of 3-axis autosampler.

Design file name	File type	Open source license	Location of the file
*RAMPS*	ZIP folder containing Processing Development Environment (*.pde) files	*CC BY 4.0*	*https://doi.org/10.17632/vfn2g8xg36.1*

The files listed in Table 3 are used in the construction of the SCARA autosampler system described in Sections 5.2.1-5.2.3.

**Table 3:** Design files for construction of SCARA autosampler.

Design file name	File type	Open source license	Location of the file
Riser *2 Needed	*STL, SLDPRT*	*CC BY 4.0*	*https://doi.org/10.17632/vfn2g8xg36.1*
*ParallelMountA_Top*	*STL, SLDPRT*	*CC BY 4.0*	*https://doi.org/10.17632/vfn2g8xg36.1*
*ParallelMountA_Front*	*STL, SLDPRT*	*CC BY 4.0*	*https://doi.org/10.17632/vfn2g8xg36.1*
*ParallelMountA_Back*	*STL, SLDPRT*	*CC BY 4.0*	*https://doi.org/10.17632/vfn2g8xg36.1*
*ParallelMountB_Top*	*STL, SLDPRT*	*CC BY 4.0*	*https://doi.org/10.17632/vfn2g8xg36.1*
*ParallelMountB_Front*	*STL, SLDPRT*	*CC BY 4.0*	*https://doi.org/10.17632/vfn2g8xg36.1*
*ParallelMountB_Back*	*STL, SLDPRT*	*CC BY 4.0*	*https://doi.org/10.17632/vfn2g8xg36.1*
*LinkA1*	*STL, SLDPRT*	*CC BY 4.0*	*https://doi.org/10.17632/vfn2g8xg36.1*
*LinkA2*	*STL, SLDPRT*	*CC BY 4.0*	*https://doi.org/10.17632/vfn2g8xg36.1*
*LinkB1*	*STL, SLDPRT*	*CC BY 4.0*	*https://doi.org/10.17632/vfn2g8xg36.1*
*LinkB2*	*STL, SLDPRT*	*CC BY 4.0*	*https://doi.org/10.17632/vfn2g8xg36.1*
*LinkP1*	*STL, SLDPRT*	*CC BY 4.0*	*https://doi.org/10.17632/vfn2g8xg36.1*
*LinkP2*	*STL, SLDPRT*	*CC BY 4.0*	*https://doi.org/10.17632/vfn2g8xg36.1*
*Elbow*	*STL, SLDPRT*	*CC BY 4.0*	*https://doi.org/10.17632/vfn2g8xg36.1*
*EndEffector*	*STL, SLDPRT*	*CC BY 4.0*	*https://doi.org/10.17632/vfn2g8xg36.1*
*LeverMain*	*STL, SLDPRT*	*CC BY 4.0*	*https://doi.org/10.17632/vfn2g8xg36.1*
*LeverAtt*	*STL, SLDPRT*	*CC BY 4.0*	*https://doi.org/10.17632/vfn2g8xg36.1*
*CapillaryClampA*	*STL, SLDPRT*	*CC BY 4.0*	*https://doi.org/10.17632/vfn2g8xg36.1*
*CapillaryClampB*	*STL, SLDPRT*	*CC BY 4.0*	*https://doi.org/10.17632/vfn2g8xg36.1*
*Fulcrum*	*STL, SLDPRT*	*CC BY 4.0*	*https://doi.org/10.17632/vfn2g8xg36.1*
*CapillaryRail*	*STL, SLDPRT*	*CC BY 4.0*	*https://doi.org/10.17632/vfn2g8xg36.1*
*CapillaryGuide*	*STL, SLDPRT*	*CC BY 4.0*	*https://doi.org/10.17632/vfn2g8xg36.1*
*Base_1*	*DXF*	*CC BY 4.0*	*https://doi.org/10.17632/vfn2g8xg36.1*
*Base_2*	*DXF*	*CC BY 4.0*	*https://doi.org/10.17632/vfn2g8xg36.1*
*Base_3*	*DXF*	*CC BY 4.0*	*https://doi.org/10.17632/vfn2g8xg36.1*

The files listed in Table 4 are used to build the PCBs and use the control software for the SCARA autosampler system, as described in the Supporting Information and *Section 6.2*.

**Table 4:** Software and electronic design files for operation of SCARA autosampler.

Design file name	File type	Open source license	Location of the file
*SCARA_code*	*ZIP folder containing Python files*	*CC BY 4.0*	*https://doi.org/10.17632/vfn2g8xg36.1*
*PiHat_ThroughHole_Control*	*DIP*	*CC BY 4.0*	*https://doi.org/10.17632/vfn2g8xg36.1*
*PiHat_ThroughHole_PowerSupply_oki-78sr*	*DIP*	*CC BY 4.0*	*https://doi.org/10.17632/vfn2g8xg36.1*
*PiHat-ThroughHole_Control*	*DHC*	*CC BY 4.0*	*https://doi.org/10.17632/vfn2g8xg36.1*
*PiHat-ThroughHole_PowerSupply_OK-78sr*	*DHC*	*CC BY 4.0*	*https://doi.org/10.17632/vfn2g8xg36.1*

## 4 Bill of materials

Table 5 describes the materials needed to construct the 3-axis autosampler, while Table 6, Table 7 describe the materials needed to construct the SCARA autosampler. All prices are current as of December 2020. Note that some hardware pieces are listed as packs of larger quantities (*e.g.*, 100), so the listed prices are slightly higher than the exact component cost that is needed for construction. However, this approach provides a cost based on the list price that would be used for purchase.

**Table 5:** Bill of Materials for the 3-Axis Autosampler.

**Component**	**Quantity**	**Cost per unit –($USD)**	**Total cost - ($USD)**	**Vendor**	**Part Number**	**Material type**
80/20 Extruded Aluminum,6 ft.	1	$ 17.79	$ 17.79	McMaster-Carr	5537T101	Metal
Angle Bracket, 4pk	2	$ 7.99	$ 15.98	Amazon	B076D9Z89G	Metal
Z-axis slide bushings	4	$ 0.69	$ 2.76	McMaster-Carr	6389K627	Metal
Leadscrew support bushing	3	$ 1.17	$ 3.51	McMaster-Carr	6389K626	Metal
Motor/Leadscrew coupling 5–8 mm, 5pk	1	$ 7.99	$ 7.99	Amazon	B073FDXHMG	Metal
X,Y-axis Leadscrew/nuts 2pk 300 mm	2	$ 14.49	$ 14.49	Amazon	B07QV4MRDD	Metal
X,Y Shafting (8 mm) 2 × 403 mm	2	$ 10.99	$ 21.98	Amazon	B07XD4FBVM	Metal
Z-Axis Leadscrew/Nut, 150 mm	2	$ 6.98	$ 13.96	Amazon	B07C8P1DWX	Metal
Z-Axis Slide Shaft 0.250″	1	$ 5.30	$ 5.30	McMaster-Carr	6061K101	Metal
X,Y Shaft supports 10 pcs. (8 needed)	1	$ 13.99	$ 13.99	Amazon	B06X94LZ33	Metal
X,Y-axis Pillow Block Bushings	8	$ 4.42	$ 35.36	McMaster-Carr	6687 K33	Metal
M5 × 10 Screws	1	$ 7.99	$ 7.99	Amazon	B07C9S7V1Z	Metal
M5 flat nuts (Pack of 100)	1	$ 16.99	$ 16.99	Amazon	B01HKMF2EE	Metal
4–40 × 0.375″	1	$ 3.10	$ 3.10	McMaster-Carr	92949A108	Metal
6–32 × 0.25″	1	$ 3.45	$ 3.45	McMaster-Carr	92949A144	Metal
6–32 × 0.5″	1	$ 3.72	$ 3.72	McMaster-Carr	92949A148	Metal
8–32 × 0.25″	1	$ 4.67	$ 4.67	McMaster-Carr	92949A190	Metal
8–32 × 0.5″	1	$ 5.54	$ 5.54	McMaster-Carr	92949A194	Metal
10–32 × 0.5″	1	$ 7.11	$ 7.11	McMaster-Carr	92949A265	Metal
M2.5 × 10 mm	1	$ 5.42	$ 5.42	McMaster-Carr	91292A014	Metal
NEMA 17 Stepper Motors, 4 pk.	1	$ 22.99	$ 22.99	Amazon	B07MP11C81	Other: Motor
Raspberry Pi 3B+	1	$ 39.99	$ 39.99	Amazon	B01CMC50S0	Other: Single-board Computer
RAMPS Board	1	$ 7.68	$ 7.68	Amazon	B06XZ46PDJ	Other: Circuit Board
RAMPS Power Supply &Barrel Plug	1	$ 15.59	$ 15.59	Amazon	B073QTNF9F	Other: Power Supply
Jumper Wires	1	$ 6.98	$ 6.98	Amazon	B01EV70C78	Composite
Stepper Drivers for Motors (5 pk.)	1	$ 9.50	$ 9.50	Amazon	B01FFGAKK8	Other: Circuit Board

**Table 6:** Bill of Materials for the SCARA Autosampler.

**Component**	**Quantity**	**Cost per unit –($USD)**	**Total cost - ($USD)**	**Vendor**	**Part Number**	**Material type**
Plywood base (for three 9″ × 14″ pieces)	1 (cut to 3)	$13.86	$13.86	Home Depot	958719	Wood: Birch plywood
Dowel pins (1/4″ dia × 5/8″)	1	$4.19	$4.19	McMaster-Carr	98381A539	Metal
Wood Glue	1	$5.97	$5.97	Home Depot	107209	Adhesive
Pine board for clamping (1″ × 8″ × 6′)	2	$3.69	$7.37	Home Depot	914827	Wood: Pine
Hitec HS422 Servo	2	$14.49	$28.98	www.servocity.com	31422S00	Other: Motor
Gobilda servo stand (2 pack)	2	$6.99	$13.98	www.gobilda.com	1804–0032-0001	Metal
4–40 × 3/4″ pan head machine screw	1	$2.39	$2.39	McMaster-Carr	90272A113	Metal
M3 × 20 mm pan head machine screw	1	$3.48	$3.48	McMaster-Carr	92005A128	Metal
M3 × 30 mm pan head machine screw	1	$4.28	$4.28	McMaster-Carr	92005A132	Metal
M4 × 20 mm pan head machine screw	1	$6.40	$6.40	McMaster-Carr	92005A232	Metal
4–40 hex nut	1	$0.89	$0.89	McMaster-Carr	90480A005	Metal
M3 hex nut	1	$1.57	$1.57	McMaster-Carr	90591A121	Metal
Hitec servo horn (included in servo kit)	2	–	–	www.servocity.com	31422S00	Other: Motor
Hitec servo horn screw (included in servo kit)	2	–	–	www.servocity.com	31422S00	Other: Motor
DE solenoid DSOL-0630-12C	1	$19.13	$19.13	Digikey	1144–1419-ND	Other: Solenoid
Comp spring 5/16″ × 1.5″ × 0.023″	1	$5.52	$5.52	McMaster-Carr	9657 K107	Metal
#0 × 1/2″ Thread-forming screw	1	$12.91	$12.91	McMaster-Carr	99461A530	Metal
4–40 × 3/4″ pan head machine screw	1	$1.80	$1.80	McMaster-Carr	90272A110	Metal
M2 × 10 mm pan head machine screw	1	$4.14	$4.14	McMaster-Carr	92005A033	Metal
M2 nut	1	$1.57	$1.57	McMaster-Carr	90591A111	Metal
13/64″ × 0.5″ binding barrel	1	$6.97	$6.97	McMaster-Carr	98002A312	Metal
13/64″ × 0.75″ binding barrel	1	$7.88	$7.88	McMaster-Carr	98002A313	Metal
Loctite 222 thread locker	1	$15.35	$15.35	McMaster-Carr	1810A27	Adhesive
M2.5 × 10 mm standoff	4	$0.62	$2.48	McMaster-Carr	95947A005	Metal
M2.5 × 5 mm pan head machine screw	1	$4.14	$4.14	McMaster-Carr	92005A061	Metal
M2.5 × 14 mm pan head machine screw	1	$6.25	$6.25	McMaster-Carr	92005A074	Metal

**Table 7:** Bill of Materials for the SCARA Control Board and Power Supply.

**Board Part**	**Quantity**	**Number**	**Cost per unit –($USD)**	**Total cost - ($USD)**	**Vendor**	**Part Number**	**Component type**
*Control Board PCB*							
	Raspberry Pi 4 Model B	1	$35.00	$35.00	Sparkfun	DEV-15446	Single-board computer
R1	221 O resistor	1	$0.10	$0.10	Digikey	221XBK-ND	Resistor
R2	562 O resistor	1	$0.10	$0.10	Digikey	562XBK-ND	Resistor
R3-4	10 k resistor	2	$0.10	$0.20	Digikey	10.0KXBK-ND	Resistor
C1-2	0.1uF ceramic cap	2	$0.42	$0.84	Digikey	490–8809-ND	Capacitor
D1-2	1 N4149 diode	2	$0.10	$0.20	Digikey	1 N4149-ND	Diode
L1	3 mm LED	1	$0.36	$0.36	Digikey	160–1958-ND	LED
Q1	transistor	1	$0.23	$0.23	Digikey	BC546ATAFSCT-ND	Transistor
U1	ADC 10 Bit	1	$1.79	$1.79	Digikey	MCP3002-I/P-ND	IC
J1	Stacking header	1	$2.95	$2.95	Digikey	1528–1783-ND	Header
U2	8 pin IC socket	1	$0.77	$0.77	Digikey	A400-ND	Socket
Power	4 pin socket	1	$1.51	$1.51	Digikey	WM14831-ND	Socket
Servo	6 pin socket	1	$1.94	$1.94	Digikey	WM4223-ND	Socket
Solenoid	2 pin RA header	1	$0.28	$0.28	Digikey	H10999-ND	Header
Buttons	4 pin RA header	1	$0.34	$0.34	Digikey	H11001-ND	Header
Pots	6 pin RA header	1	$0.46	$0.46	Digikey	H11003-ND	Header
Solenoid	2 pin plug	1	$0.13	$0.13	Digikey	H3781-ND	Plug
Buttons	4 pin plug	1	$0.16	$0.16	Digikey	H3783-ND	Plug
Pots	6 pin plug	1	$0.16	$0.16	Digikey	H3785-ND	Plug
Pins	crimping pins	50	$0.066	$3.30	Digikey	H3828-ND	Pins for plugs
K1	5 V relay G5V	1	$3.18	$3.18	Digikey	Z108-ND	Relay
S1	DC solenoid	1	$17.60	$17.60	Digikey	1144–1404-ND	Solenoid
J1	Joystick	1	$3.95	$3.95	Digikey	1568–1526-ND	Joystick
P1	12 V 60 W AC/DC	1	$17.85	$17.85	Digikey	Q1185-ND	Power supply

*Power Supply PCB*							
R1-2	560 O resistor	2	$0.10	$0.20	Digikey	562XBK-ND	Resistor
R3-4	1 k resistor	2	$0.10	$0.20	Digikey	1.00KXBK-ND	Resistor
R5	2.4 k resistor	1	$0.10	$0.10	Digikey	2.37KXBK-ND	Resistor
C1-3	22uF ceramic cap	3	$1.82	$5.46	Digikey	490–14507-ND	Capacitor
D1-2	1 N4149 diode	2	$0.10	$0.20	Digikey	1 N4149-ND	Diode
D3-5	3 mm LED	3	$0.36	$1.08	Digikey	160–1958-ND	LED
U1-2	5 V voltage reg	2	$4.30	$8.60	Digikey	811–2692-ND	IC
F1-2	Fuseholder (1/2)	4	$0.13	$0.52	Digikey	F4189-ND	Hardware
J1	stacking header	1	$2.95	$2.95	Digikey	1528–1783-ND	Header
S1	slide switch	1	$4.08	$4.08	Digikey	360–2728-ND	Hardware
P1	power jack	1	$0.74	$0.74	Digikey	CP-202AH-ND	Hardware
Power	4 pin header	1	$0.49	$0.49	Digikey	H3617-ND	Header

**Table 8:** Movement characteristics of the two autosampler designs.

	**3-Axis Autosampler**			**SCARA Autosampler**	
	***X (in.)***	***Y (in.)***	***Z (in.)***	***X (in.)***	***Y (in.)***
***Accuracy***	±0.001	±0.002	±0.001	±0.008	±0.023
***Repeatability***	±0.006	±0.007	±0.005	±0.026	±0.012

*Additional tools that will be needed for the 3-axis autosampler system include:*

Power mitre saw (or similar), rotary cutting tool (or similar), hex key allen wrench set, screwdriver set, standard tap & die set, and a 3D printer. For this design, an Ultimaker 3 with Ultimaker PLA filament (Ultimaker B.V., Utrecht, Netherlands) was used. Most components were printed with a 100% infill and 0.1 mm layer height using the grid pattern infill design. Larger parts (‘*x_carriage_frame*’ and ‘*well_plate_holder*’) were printed with 60% infill and 0.15 mm layer height using the same infill design.

*Additional tools that will be needed for the SCARA autosampler system include:*

Trigger clamps (6″), paper towels, sandpaper, laser cutter, woodcutting saw, screwdriver set, wrench or pliers, and a soldering station, and a 3D printer. For this design, a Monoprice Mini V2 with Monoprice PLA filament (Monoprice, Inc., Brea, CA) was used to print all parts with default settings: 22% infill, 0.1 mm layer height, and a grid pattern infill design.

For both designs, the reported print settings were primarily based on default settings, so similar procedures on other 3D printers would likely be suitable to create the necessary parts.

## 5 Build instructions

### 5.1 Build instructions for 3-Axis system

#### 5.1.1 Initial preparation for 3-Axis system

##### 5.1.1.1 Sizing of commercial parts

**Fig. 3:**
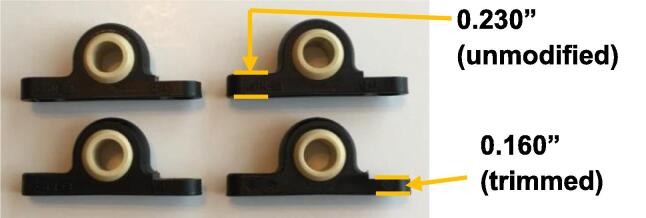
Modifications needed on four X-axis slide bushings.

Make the frame portions from the 6′ long piece of T-slotted framing extrusion (PN# 5537T101) by cutting 2 pieces to a length of 8.75″, 3 pieces to a length of 7.50″, and 2 pieces to a length of 6.50″. Cutting the T-slotted framing can be accomplished with a hacksaw or other hand tool, but this is one area where a square cut can make assembly easier. A power mitre saw is best, but if one is not accessible some vendors will make the cuts for a small fee.
Make the Z-axis rails by cutting the ¼″ × 8″ long shaft (PN# 6061K101) into 2 pieces 3.65″ long. This can be accomplished with an angle grinder or rotary tool with a metal cutting wheel. A hacksaw or similar will not work as the shafting is very hard (60 HRC).
Make the X, Y-axis rails by cutting the 8 mm shafting (PN# B07XD4FBVM) into 4 pieces 8.90″ long.
Make the X, Y, Z-axis lead screws from the commercial parts (PN# B07QV4MRDD, PN# B07C8P1DWX). Two 9.25″ length pieces of B07QV4MRDD are needed for the X-axis, two 8.40″ length pieces of B07QV4MRDD are needed for the Y-axis, and two 3.40″ length pieces of B07C8P1DWX are needed for the Z-axis. The lead screws are softer material, and therefore they may be cut with an angle grinder, rotary cutting tool, or hack saw.
Reduce the four X-axis slide bushings (PN# 6687K33) to 0.160″ thickness on one side to increase room for lead screw clearance (Fig. 3). This process can be accomplished with a file or rotary cutting tool.

##### 5.1.1.2 Modifications to 3D-Printed parts

1. Prepare the well plate holder by threading indicated holes into *well_plate_holder.STL* with an appropriate tap (see Fig. 4).

**Fig. 4:**
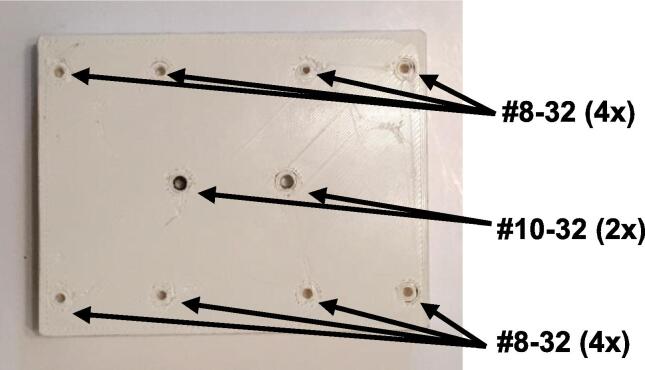
Thread sizes for tapped holes in bottom of well plate holder piece.
2. Prepare the X-axis carriage by threading the indicated holes into *x_carriage_frame.STL* with an appropriate tap (see Fig. 5).

**Fig. 5:**
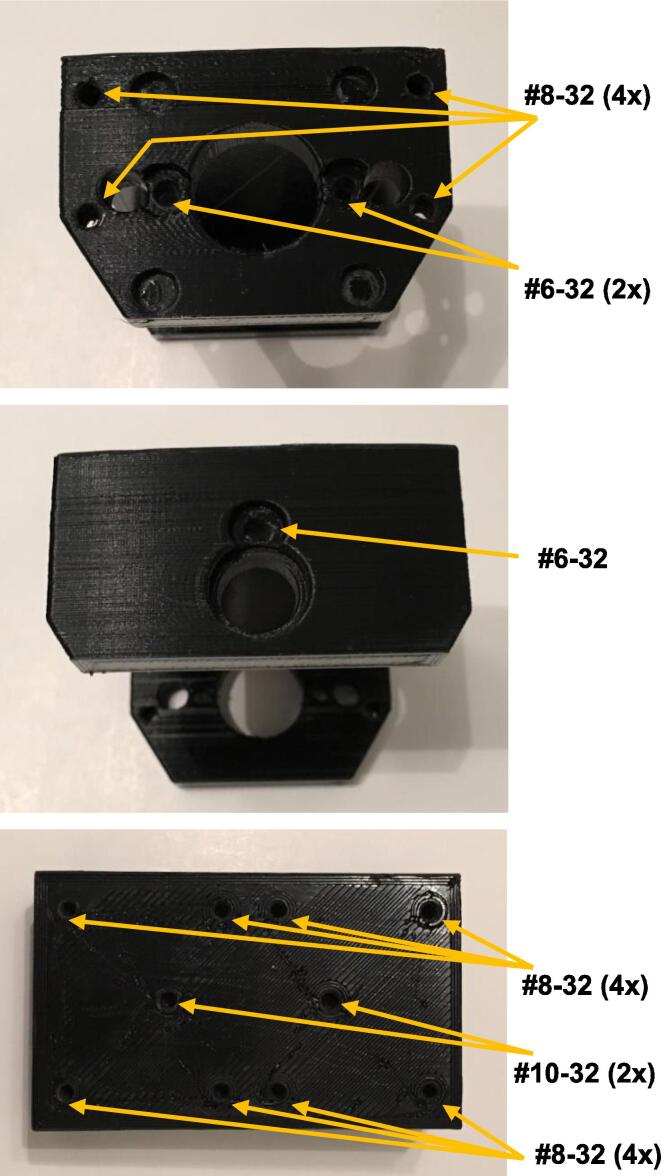
Thread sizes for tapped holes in X-axis carriage piece.
3. Prepare the Z-axis carriage by threading the indicated holes into *process_interface_carriage.STL* with an appropriate tap (see Fig. 6).

**Fig. 6:**
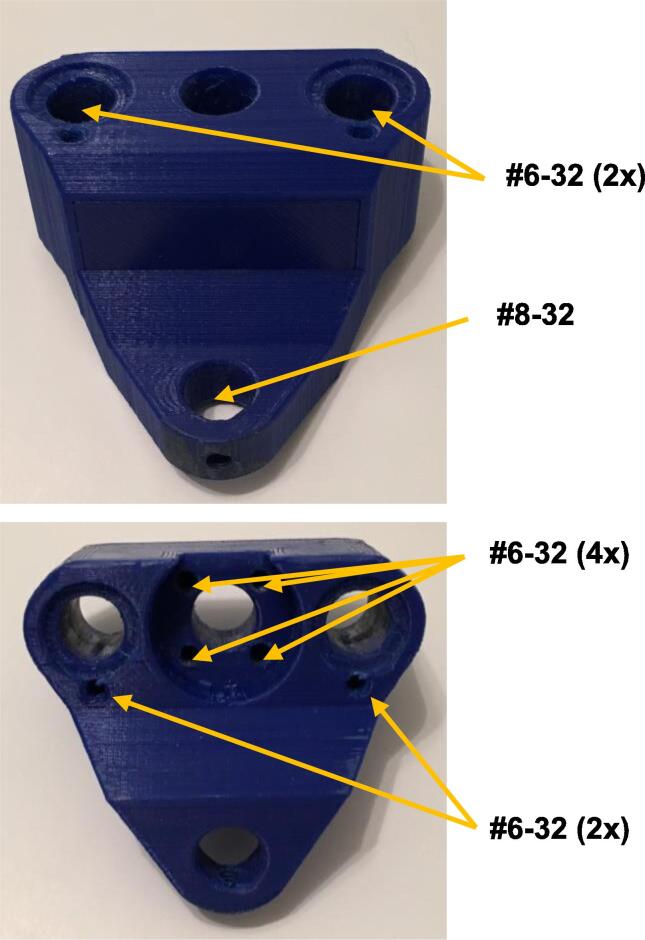
Thread sizes for tapped holes in Z-axis carriage pieces.
4. Prepare the X, Y-axis lead screw supports (*_axis_leadscrew_support.STL)) and screw nut housings (leadscrew_nut_housing_*_axis.STL) by threading the indicated holes into each piece with an appropriate tap (see Fig. 7).

**Fig. 7:**
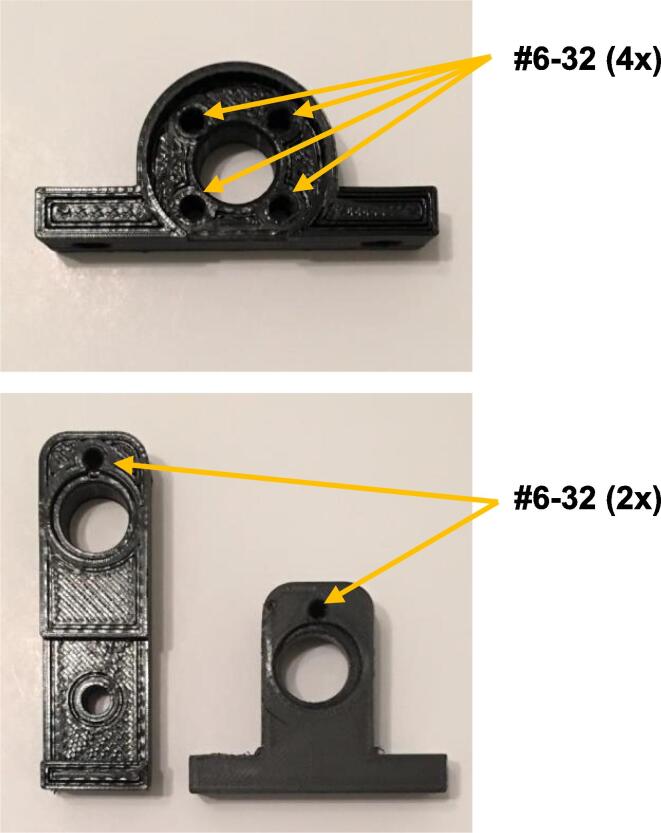
Thread sizes for tapped holes in X, Y-axis screw nut housings (top) and lead screw support pieces (bottom) and screw nut housings.
5. Finish the capillary holder pieces (*capillary_elec_insert.STL*) by filing a small groove for the capillary into one piece. While both halves have a conical feature to aid insertion, it is advisable to only file one side as shown in Fig. 8.

**Fig. 8:**
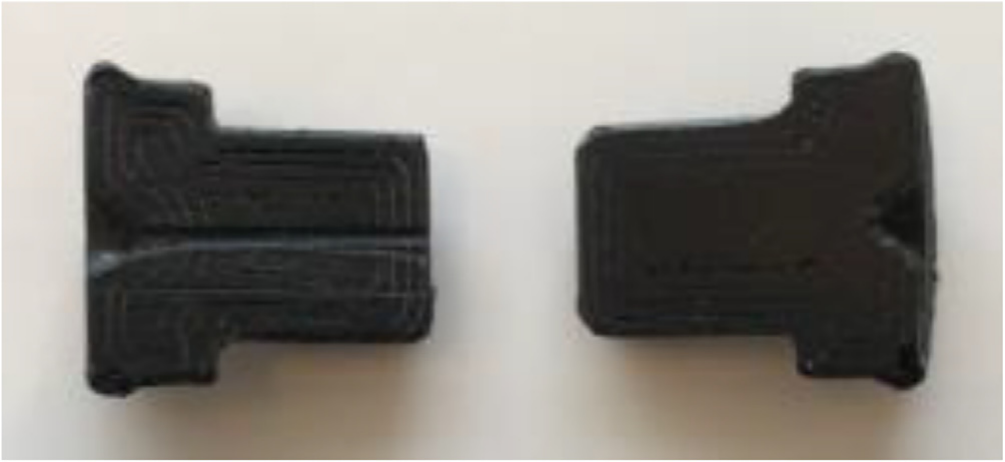
Filed groove in one piece of capillary holder module.

#### 5.1.2 Sub-Assemblies for 3-Axis System

1. Press-fit the lead screw support bushings (PN# 6389K626) into the X, Y-axis lead screw supports (Fig. 9). Secure the bushings with bushing retaining button head cap screw #6-32 × 0.25″. To reduce number of vendors, a 0.3125″ bushing was specified while the lead screw is actually 0.315″. We enlarged bushing PN# 6389K626, but an alternate 8 mm bushing can also be purchased.

**Fig. 9:**
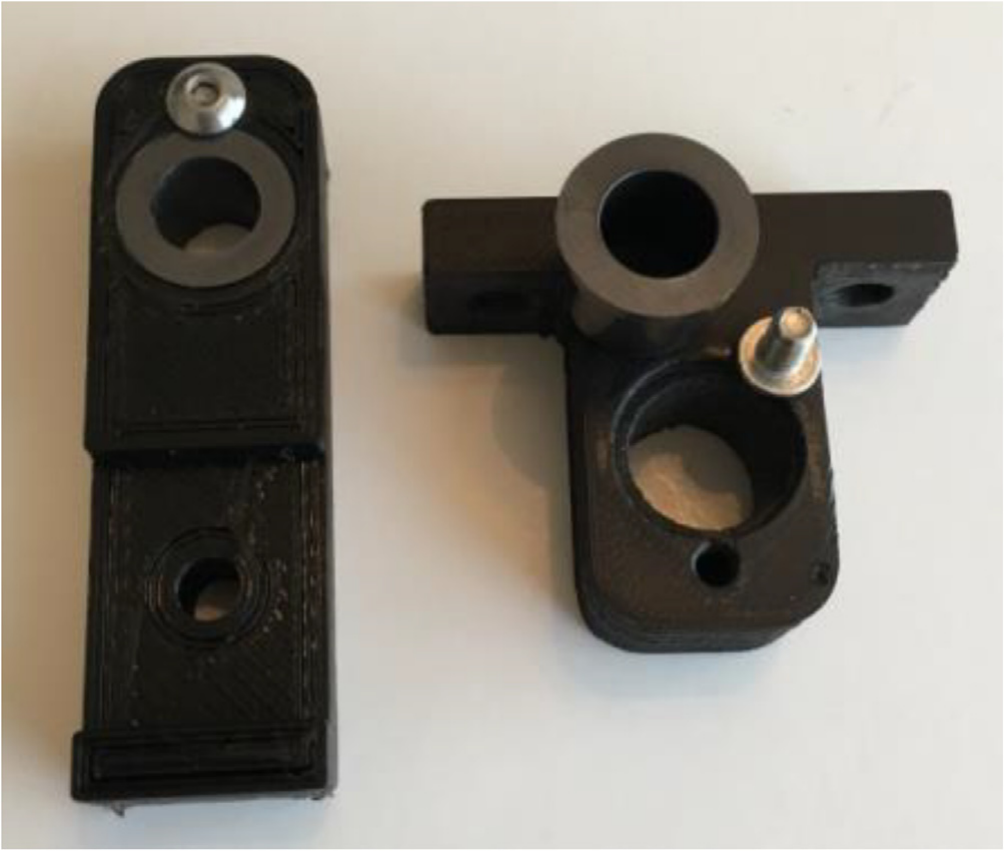
Press-fit and secured bushing into X, Y-axis lead screw supports.
2. Bolt the X,Y-axis lead screw nuts (included with B07QV4MRDD) into their housings using four #6–32 × 0.5″ button head cap screws (Fig. 10). The lead screw nuts will need to have a portion of the flange removed to be congruent with the holder body. This may be done with a file or rotary tool.

**Fig. 10:**
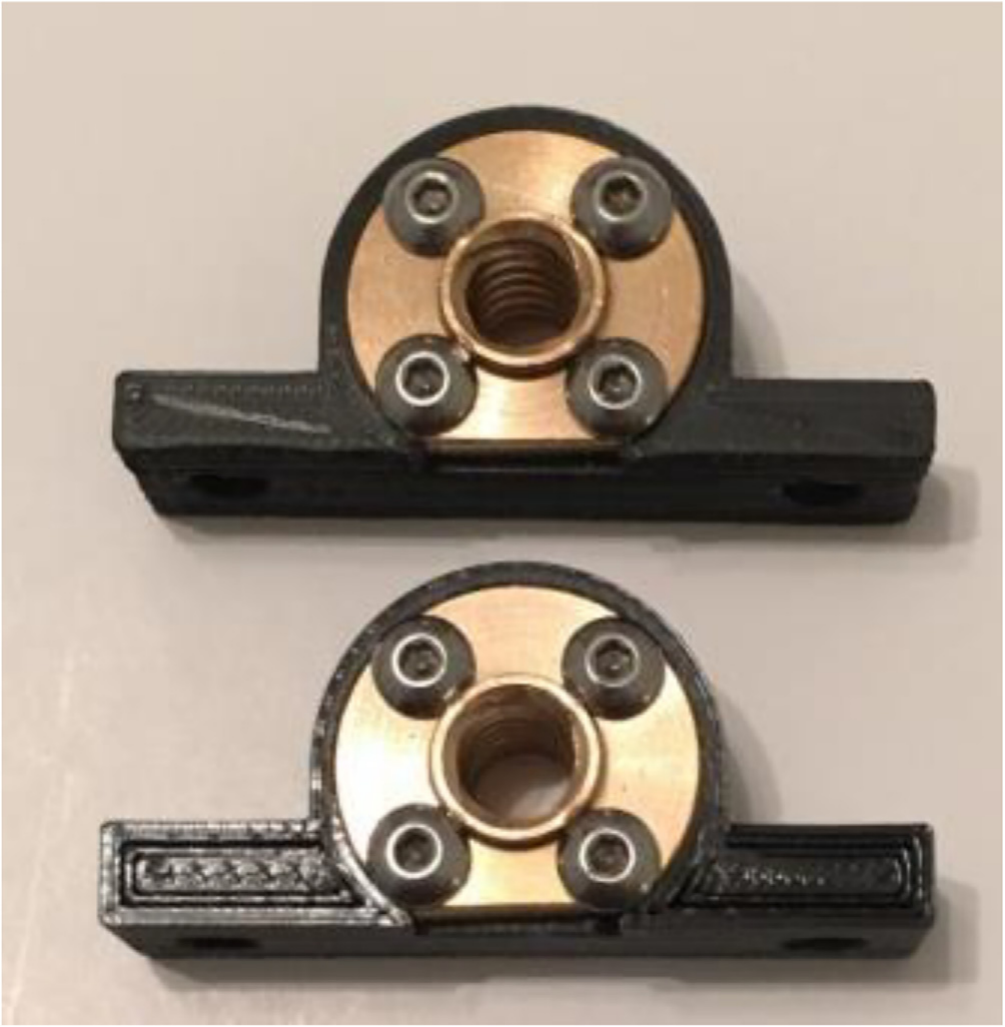
Secured X,Y-axis lead screw nuts in housings.
3. To attach the Y-axis carriage onto well plate holder (Fig. 11), bolt four Y-axis pillow block bushings (PN# 6687K33) to the corners of the well plate holder using two #8–32 × 0.5″ button head cap screws each (8 total), but do not completely tighten screws to improve positioning later in construction. Attach one of the X,Y-axis lead screw nut holder from the previous step to the center of the well plate holder using two #10–32 × 0.5″ button head cap screws, again without completely tightening them.

**Fig. 11:**
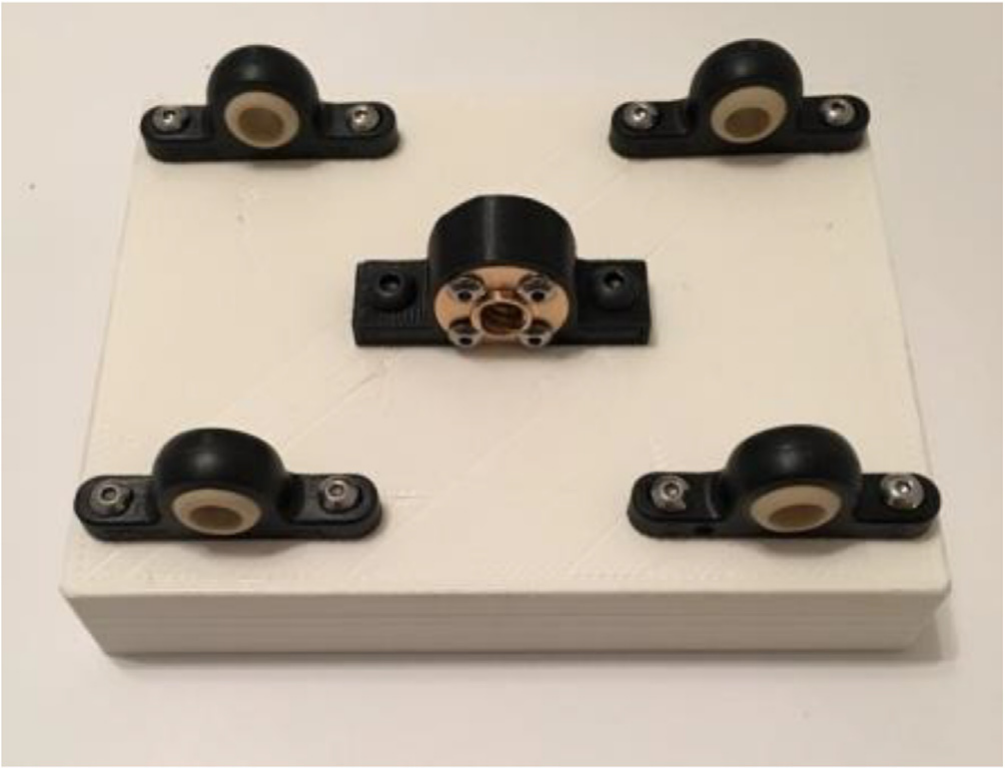
Bottom of well plate holder with attached Y-axis carriage components.
4. To form the Z-axis carriage (Fig. 12), press-fit two Z-axis slide bushings (PN# 6389K627) into the bottom of the ‘Process Interface Carriage’ component. Bolt down the Z-axis lead screw nut (included with B07C8P1DWX) on this same side with #6–32 × 0.5″ button head cap screws. Turn the carriage piece over and press-fit the two remaining Z-axis slide bushings (PN# 6389K627) into place. Here, the retainer screws for the Z-axis slide bushings should not be necessary due to the retention of the press-fit.

**Fig. 12:**
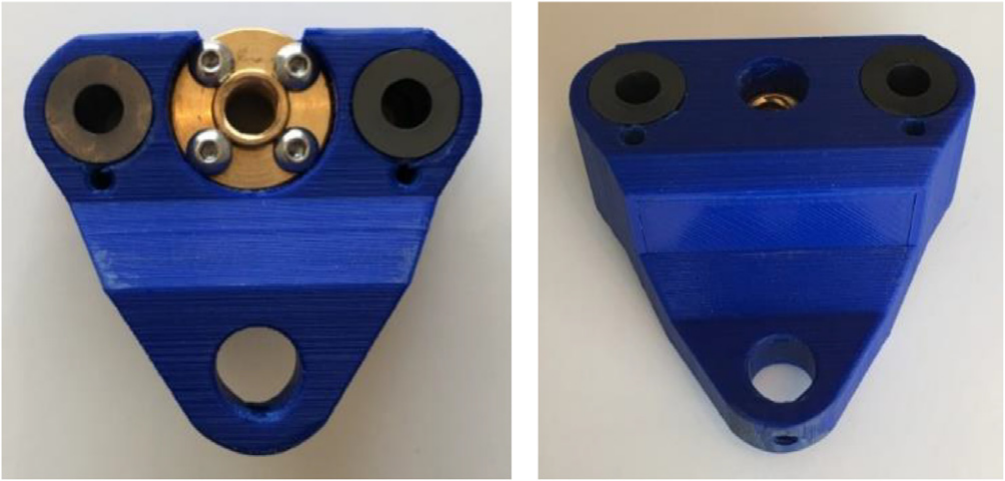
Bottom (left panel) and top (right panel) of Z-axis carriage component.
5. To prepare the X-axis carriage that also holds the Z-axis carriage, press-fit the Z-axis lead screw support bushing (PN# 6389K626) into the top of the X-axis carriage piece and secure it with bushing retaining button head cap screw #6–32 × 0.25″. Bolt the remaining X,Y-axis lead screw nut holder (Fig. 10) to the center of the back of the X-axis carriage piece using two #10–32 × 0.5″ button head cap screws, again without completely tightening them. Then, connect the four X-axis pillow block bushings (PN# 6687K33) in each corner using #8–32 × 0.5″ button head cap screws for each outer hole and #8–32 × 0.375″ button head cap screws for each inner hole. The inner hole should be the reduced 0.160″ thick portion of the bushing (Fig. 13). Again, do not completely tighten the screws due to positioning requirements later in the build.

**Fig. 13:**
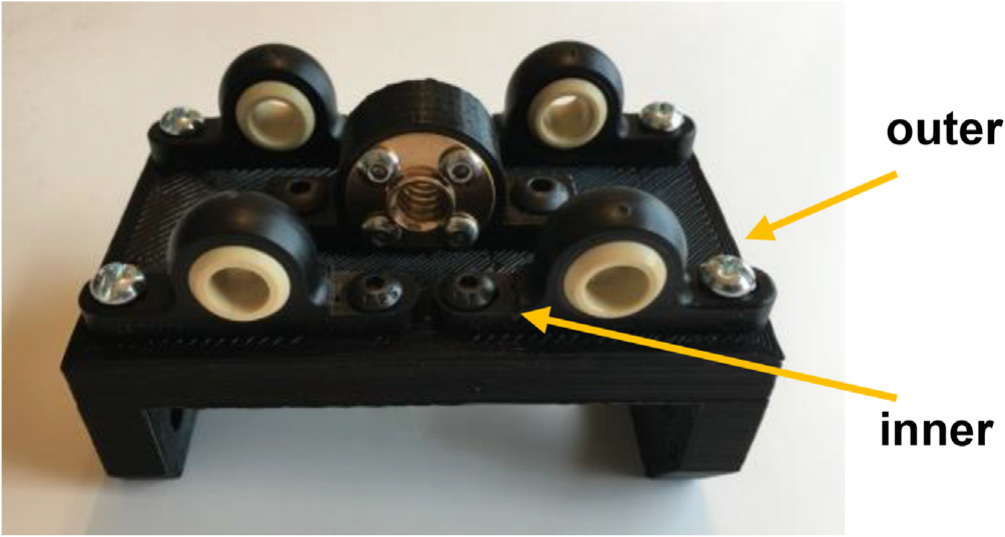
Back side of X-axis carriage with X-axis lead screw holder and bushings.
6. Combine the X-axis and Z-axis carriage components by inserting the two Z-axis slide rails (PN# 6061K101) through both components. Secure them in place with a #8–32 × 0.250″ button head cap screws for each rail on the bottom of the X-axis carriage piece. The portion of the Z-axis carriage designed to extend away from the lead screw should point toward the bottom of the combined piece (Fig. 14).

**Fig. 14:**
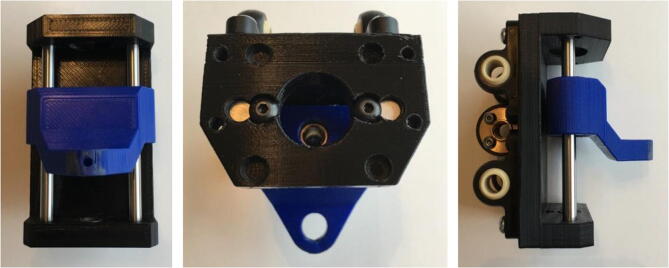
From (L-R): the front, bottom, and side of the combined X,Z-axis carriage.
7. To create the X,Y,Z-axis drive assemblies, attach the motor/lead screw couplings (PN# B073FDXHMG) to each of the three NEMA-17 axis drive motors, leaving approximately 0.325″ of the motor shaft open below the bottom of the coupling (Fig. 15).

**Fig. 15:**
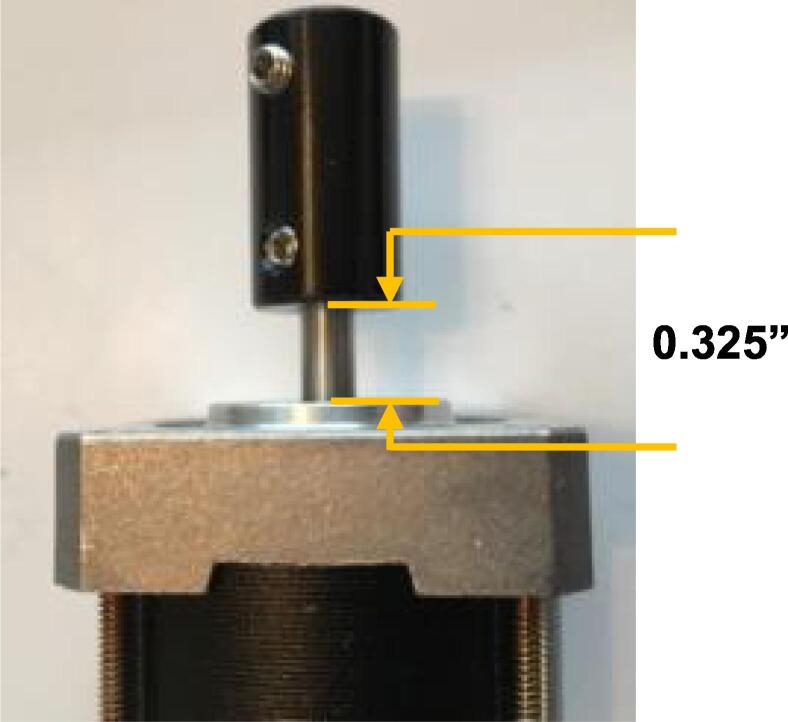
Drive assembly with motor/lead screw coupling.
8. Bolt the three printed NEMA-17 mount plates (two of *nema_17_xy_mount.STL* and one of *nema_17_z_mount.STL*) using four #4–40 × 0.375″ button head cap screws for each plate (Fig. 16). Then, secure the lead screws to each motor using the couplings.

**Fig. 16:**
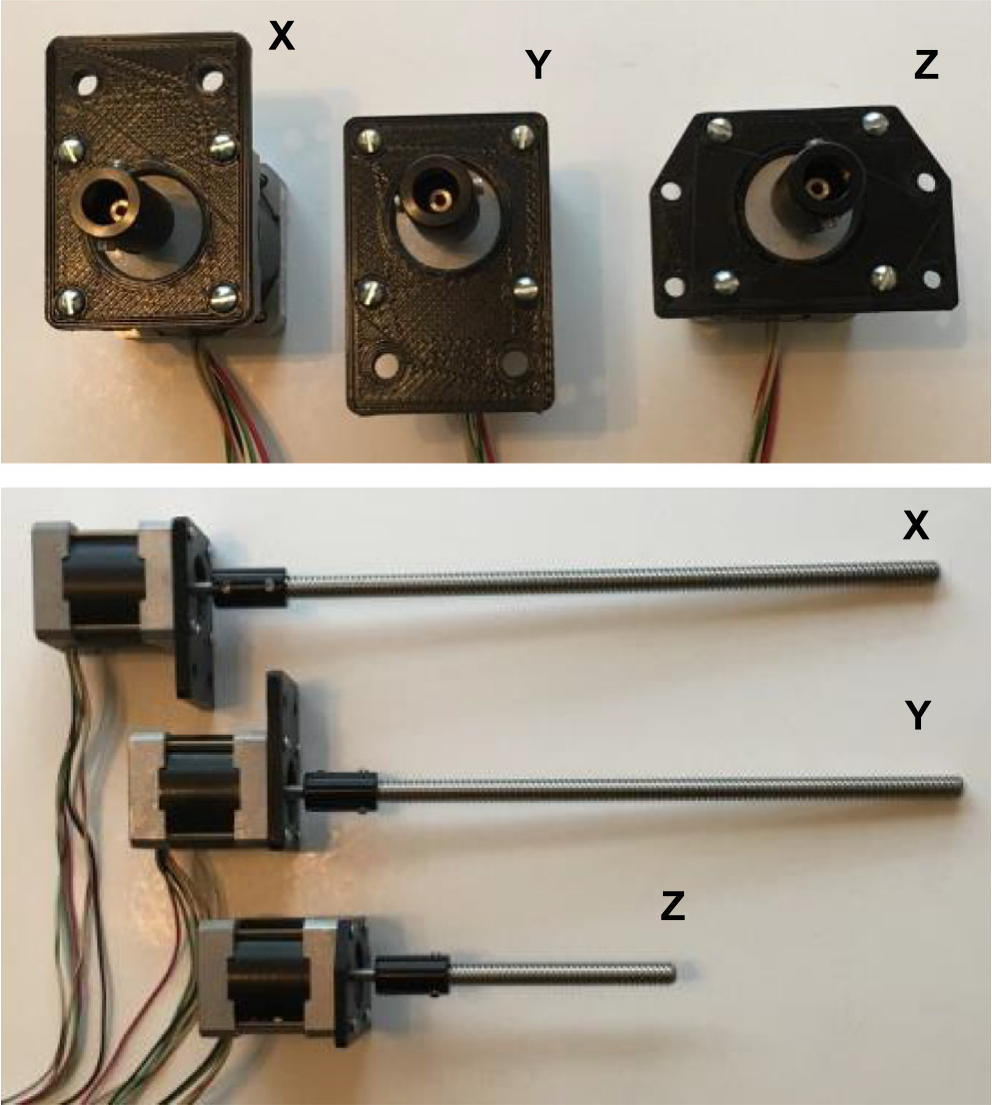
Connection of NEMA-17 motors with connected plates (top panel, front view) and lead screws (bottom panel, side view).

#### 5.1.3 Frame assembly for 3-Axis system

A clean, flat work surface makes frame assembly far easier. Calipers make an excellent layout instrument as they can be set to precise lengths and used to score lines directly on the framing. Framing / Fastener type: There are two distinct types of 20 mm square framing available: 80/20 and PZRT. We have selected 80/20 here as it is generally more available, but compatible fasteners tend to be more expensive. PZRT is more difficult to acquire in small lots, but there tends to be a wider selection of lower cost fasteners. Both formats offer standard and twist-in fasteners. For the purposes of this design, the M5 screws (PN# B07C9S7V1Z) and M5 flat nuts (PN# B01HKMF2EE) are used for standard connections. Twist-in fasteners are convenient as they can be placed into a section of framing even if the end is not open (to slide the fastener in). Whichever version is selected, the most important thing is to ensure that the frame and fasteners are compatible with each other.

1. To begin assembling the base of the frame, tighten shaft supports on the left side of the 7.5″ 80/20 frame pieces, with the left edge of each support being 0.850″ from the end of the frame piece (Fig. 17). Add in the Y-axis lead screw support on the front frame piece (without fully tightening the screws) and then place an additional shaft support on each piece (again, not fully tightened). Finally, on the back piece, slot in two additional M5 flat nuts before the frame base is completed, as they will be needed to complete the Y-axis assembly and cannot be added after the next step.

**Fig. 17:**
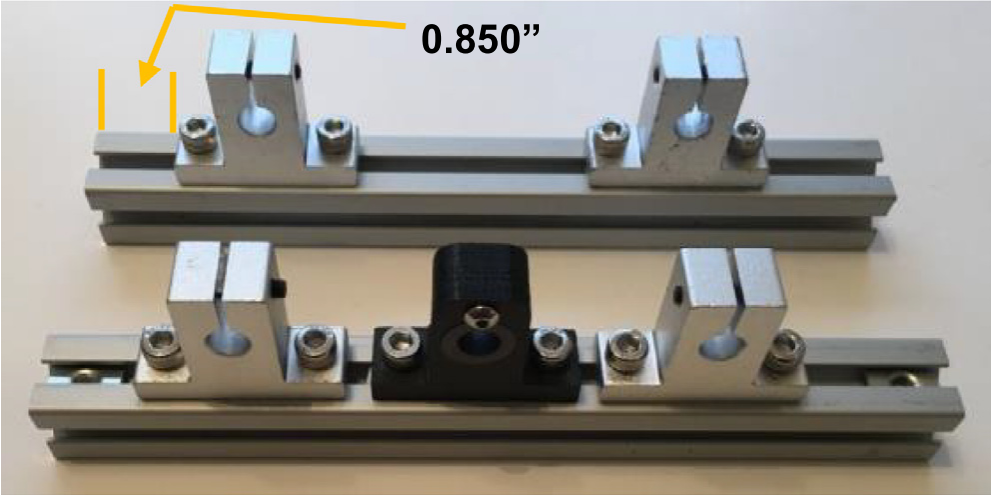
Front and back pieces of base frame.
2. Use four angle brackets (PN# B076D9Z89G) to connect the two 8.75″ pieces of 80/20 onto the two pieces prepared above, keeping the left aligned shaft supports on the left side. Attach two more angle brackets in a perpendicular position (1.180″ from the back of the base frame) that will be used to hold the frame bridge in place (Fig. 18).

**Fig. 18:**
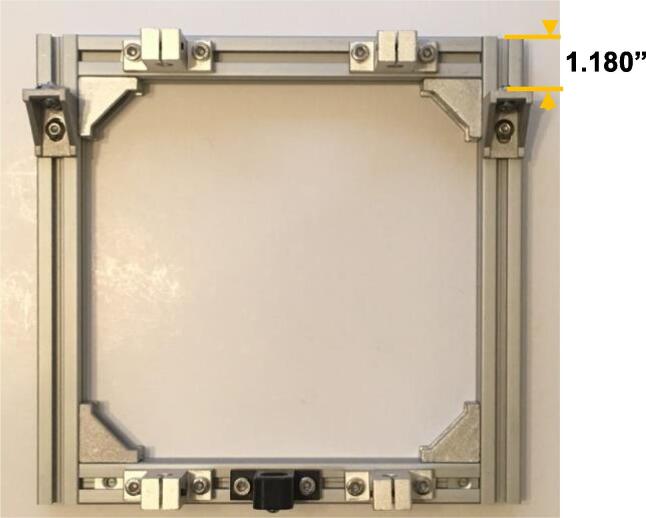
Top down view of the completed base frame.
3. To assemble the frame bridge, prepare the two 6.50″ length pieces of 80/20 extruded aluminum with angle brackets, shaft supports, and the X-axis lead screw support at the positions shown in Fig. 19. Connect the two pieces using the remaining 7.50″ length aluminum piece as a cross-beam. Fully tighten the top shaft support and angle brackets, but do not completely tighten the other screws to enable positioning later in the build.

**Fig. 19:**
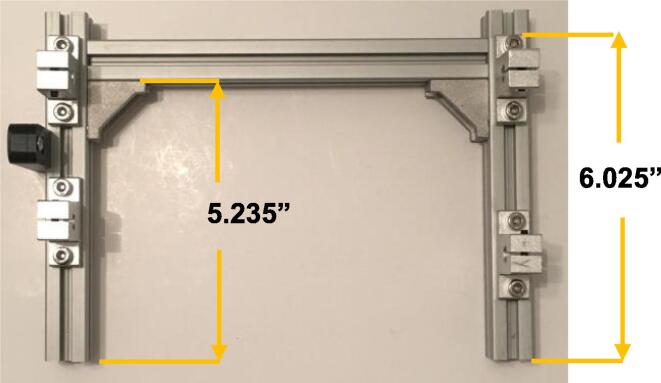
Completed frame bridge, with positions for angle brackets (to hold cross beam) and position of supports.
4. Attach the frame bridge to the base frame using the angle brackets on the top of the base frame (Fig. 20).

**Fig. 20:**
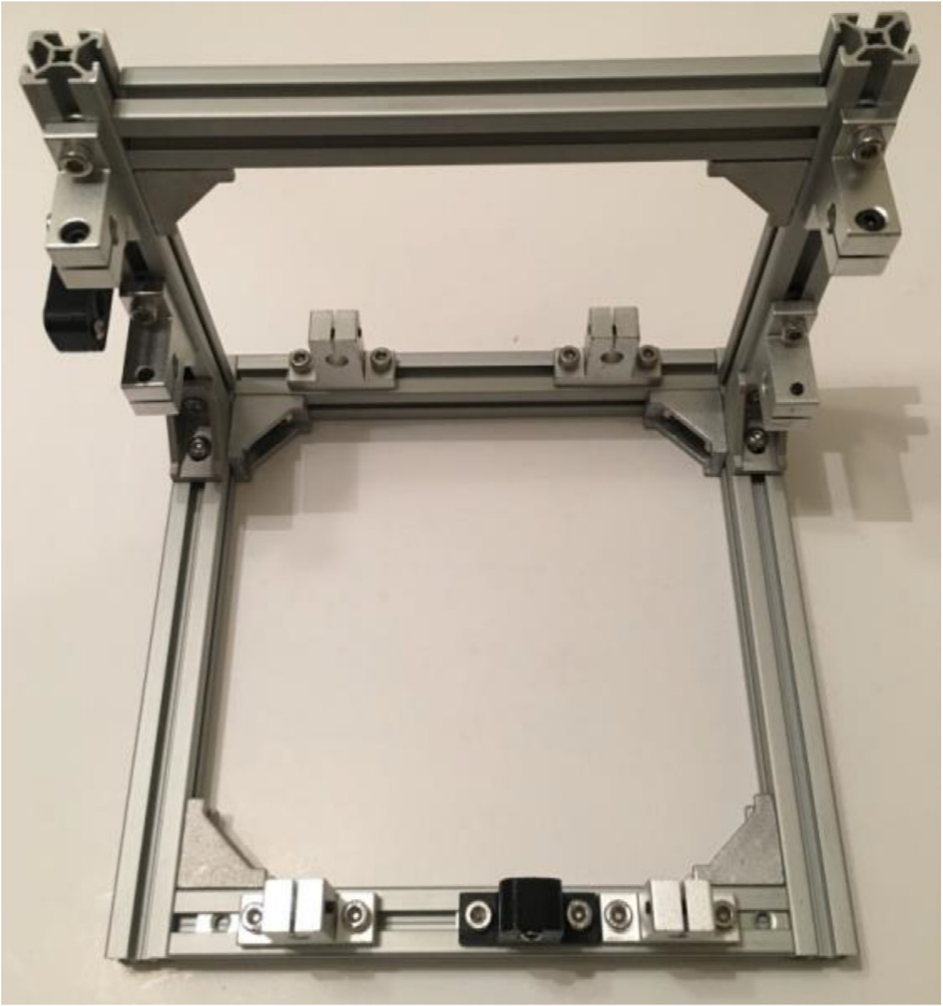
Completed frame for the 3-Axis Autosampler System.

#### 5.1.4 Axis drive Installation for 3-Axis system

1. Install the Y-axis drive by inserting the two Y-axis slide rails through the shaft supports and the bushings on the Y-axis carriage (bottom of well plate holder) as shown in Fig. 21. Start with the left side support that was fully tightened, and then slide in the right rail. Slide the Y-axis carriage to the forward-most position and tighten the remaining screws in the right front shaft support. Repeat this process with the Y-axis carriage in the rear-most position. Then, repeat the entire process for the X-axis carriage. At this point, ensure that both axis slides are moving freely with uniform resistance along each travel path. Once it is confirmed that there is no binding along the travel paths, tighten the screws in the bushings on both carriages.

**Fig. 21:**
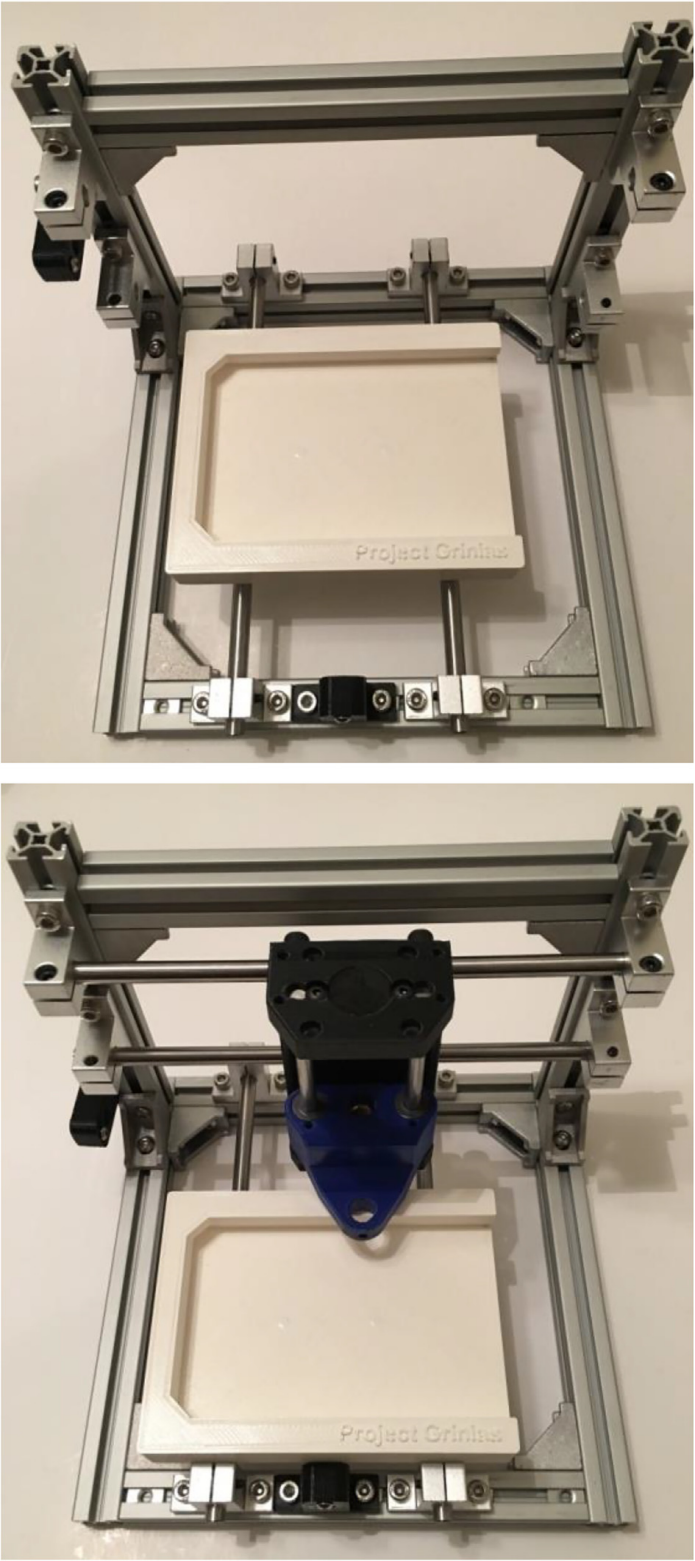
Installation of slide rails for Y-axis (top) and X-axis (bottom).
2. Lean the auto-sampler onto its back side and thread the X-axis lead screw through its lead screw nut until it is fully inserted (Fig. 22). Using two M5 screws, loosely secure the X-axis NEMA-17 motor (PN# B07MP11C81) to the right of the frame using the two remaining flat nuts that were previously put in place. Rotating the lead screw by hand, move the X-axis carriage to the right-most position and fully secure the motor to the frame. Then, manually move the Y-axis carriage to the left-most position and tighten the lead screw support mounting screws. Repeat the process for the Y-axis lead screw after returning the frame to its standard, upright position (Fig. 22). During positioning, ensure that any resistance that is not electrical in nature, as stepper motors provide rotational resistance when their lead wires are shorted together.

**Fig. 22:**
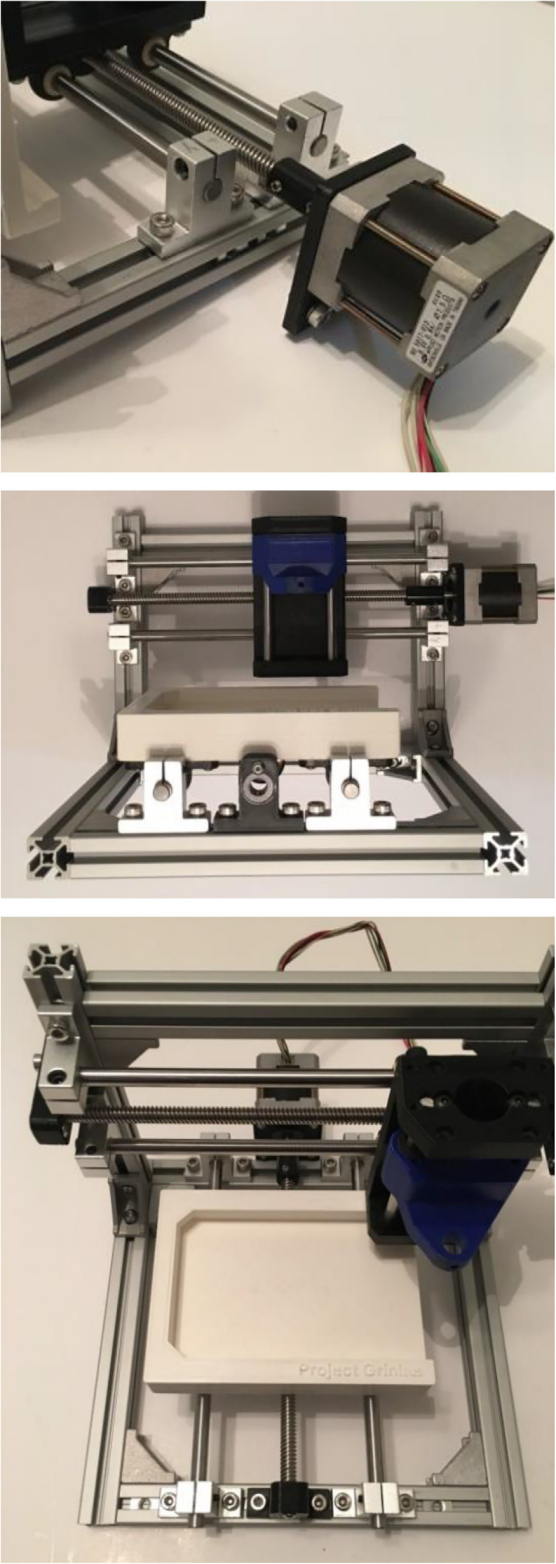
Installation of X-axis motor with frame placed on back (top), installed X-axis motor with frame in upright position (center), and installed Y-axis motor (bottom).
3. Repeat a similar process for the installation of Z-axis drive by threading the Z-axis lead screw through its lead screw nut already installed in the X-axis carriage frame (Fig. 23). Then, hold the mounting plate down to the X-axis carriage with four #8–32 × 0.5″ button head cap screws.

**Fig. 23:**
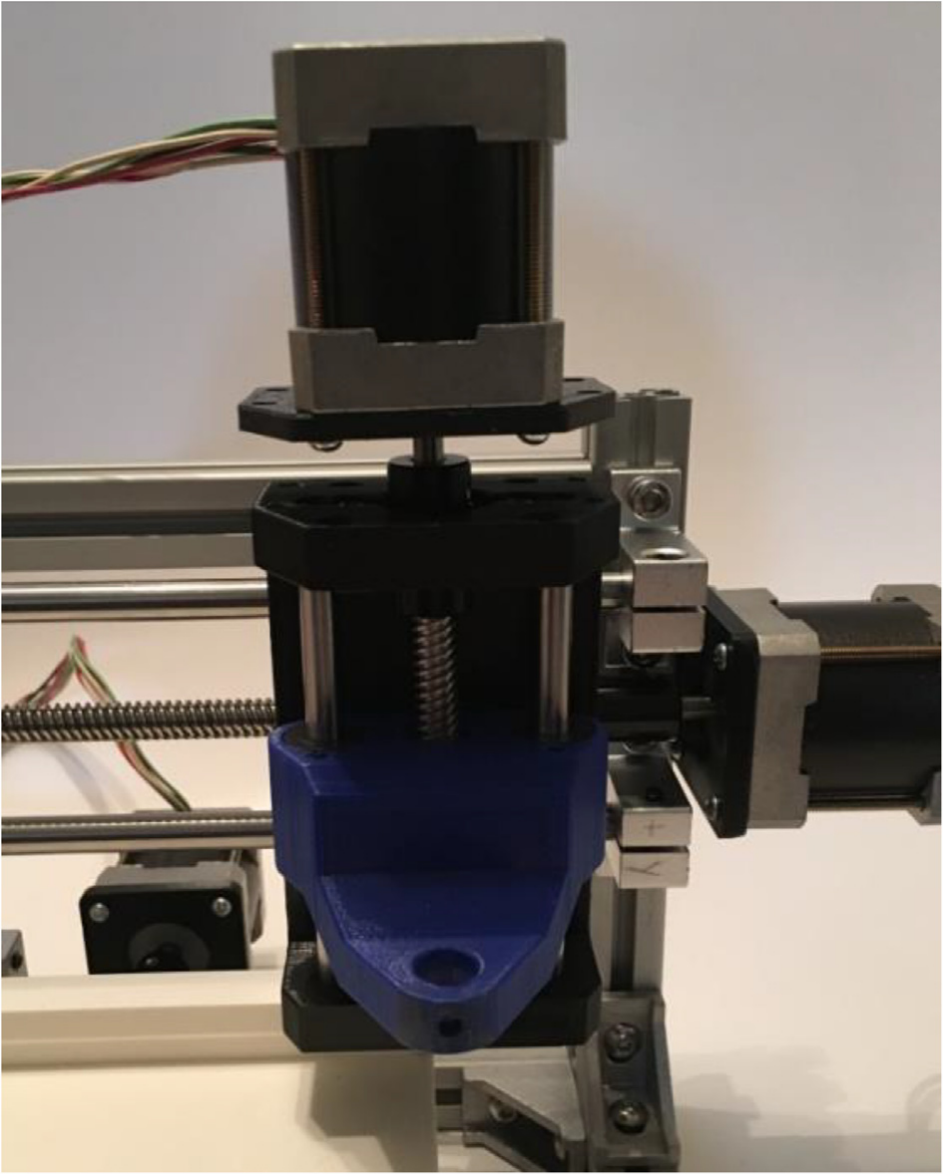
Installation of Z-axis motor on top of X-axis carriage frame.
4. Inspect the completed 3-Axis Autosampler System construction.

*Note: Simple modifications can be made with additional M5 screws and flat nuts, 3D printed mounts, and additional cuts of 80/20 extruded aluminum to mount electronic parts to the back of the frame or install a mount for a Raspberry Pi-compatible touchscreen interface on the front of the frame to further integrate components of the system.

#### 5.1.5 Raspberry Pi connections and software installation for 3-Axis system

1. Insert two pieces of 18-gauge wire into the ends of a barrel plug splitter for eventual connection to the power supply. ***Make sure the supply is not plugged in, as the exposed wires can be dangerous***. Ensure that the wires are properly secured into the barrel plug splitter (included with PN# B073QTNF9F), then connect them to the screw terminal on the RAMPS board (PN# B06XZ46PDJ) as shown in Fig. 24.

**Fig. 24:**
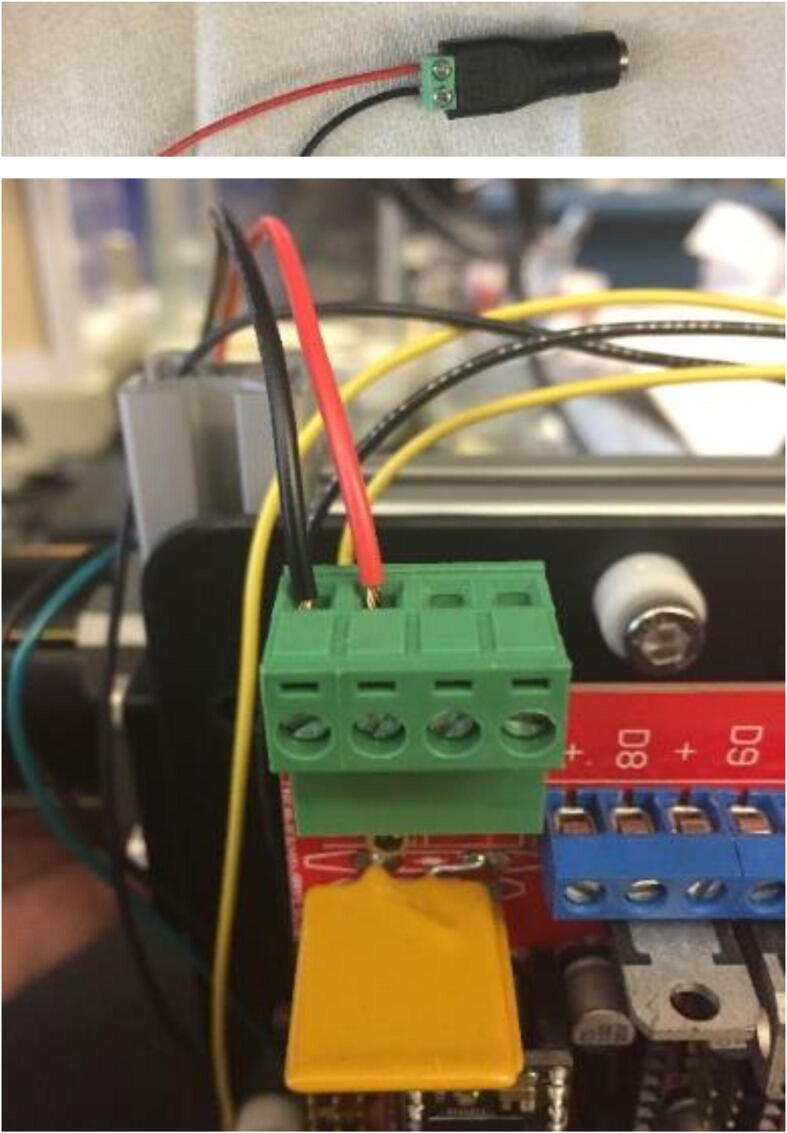
Connection of 18-gauge wires to barrel plug splitter (top) and insertion of wires into screw terminal on RAMPS board (bottom).
2. With the power supply not plugged in, place each stepper motor driver (PN# B01FFGAKK8) into the RAMPS board (Fig. 25). These drivers often have small edges that overlap, which can be fixed by gently sanding the sides of each driver until they slide in easily.

**Fig. 25:**
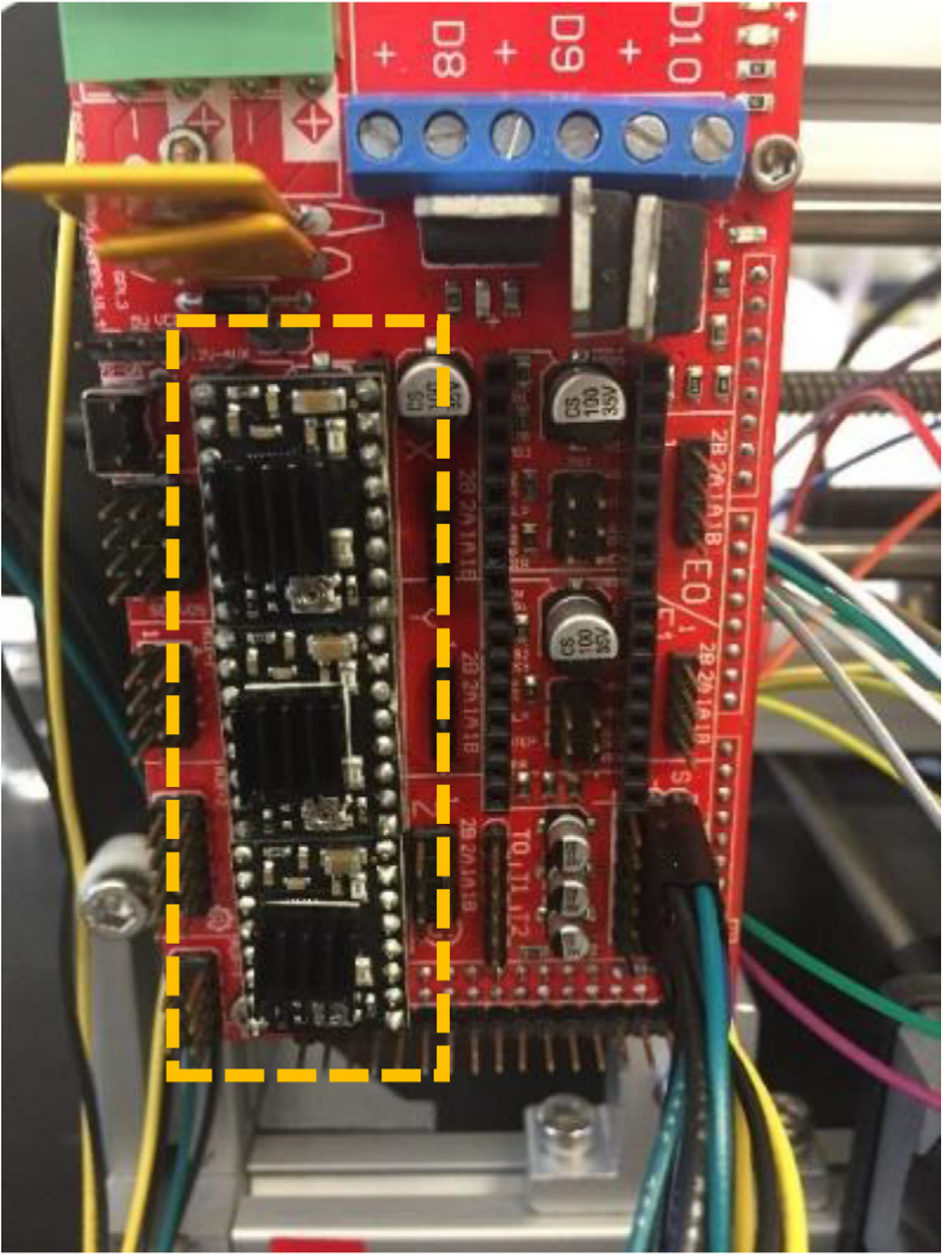
Position of three motor drivers in the RAMPS board (outlined in dashed yellow line). (For interpretation of the references to color in this figure legend, the reader is referred to the web version of this article.)
3. Connect the RAMPS board to the Raspberry Pi 3B GPIO pins using the pin diagram shown in Fig. 26. Plug in the Raspberry Pi to an appropriate power supply so that the 5 V GPIO pin output is delivering 5 V to the RAMPS board.

**Fig. 26:**
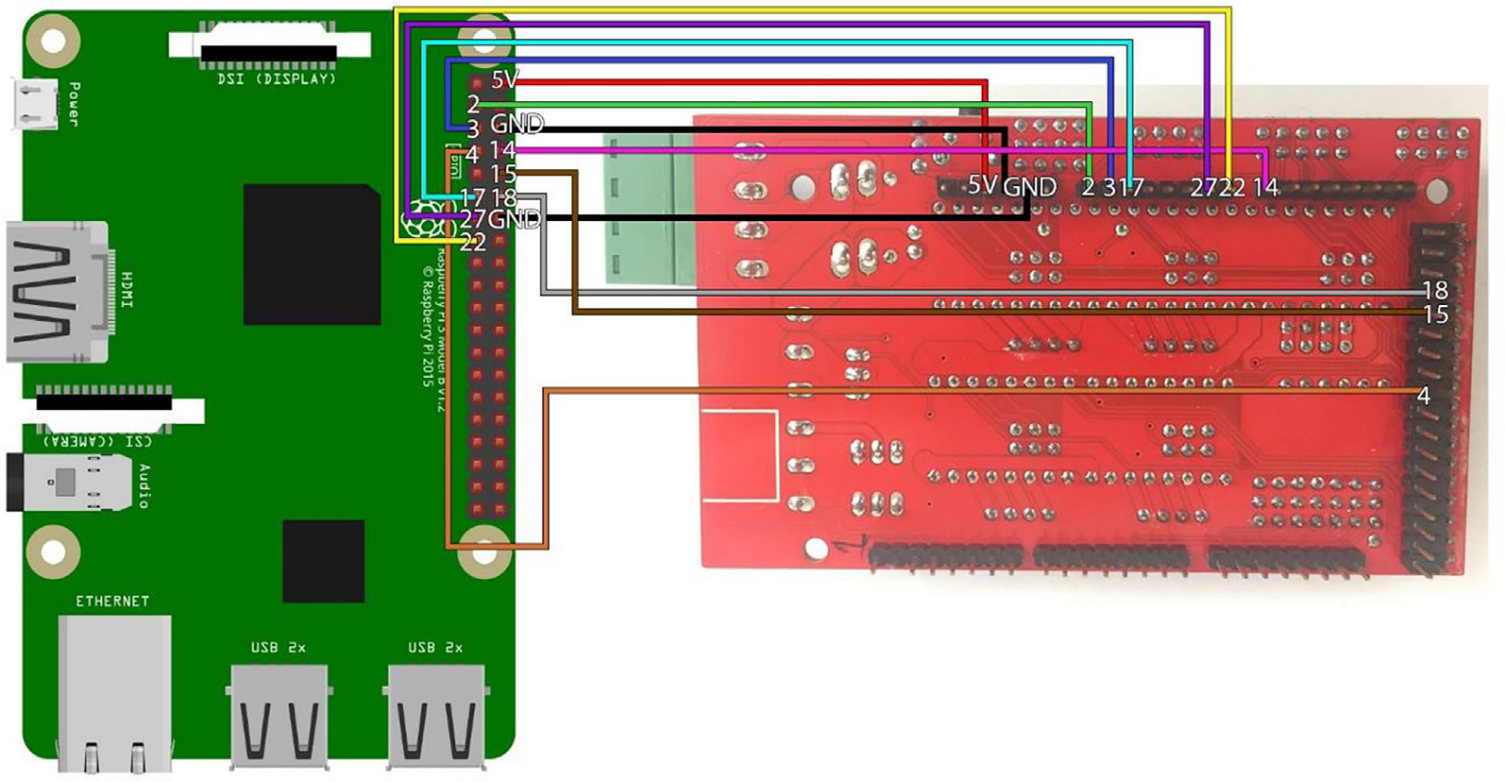
Connection of Raspberry Pi 3B and RAMPS board using combined Fritzing schematic [30] and board photograph.
4. To set the reference voltage (*V_ref_*) for the stepper motor drivers, calculate an appropriate value based on the maximum current (*I_max_*) for the motor using the following equation:

(1) $$V_{\mathrm{ref}}=0.544\times I_{\max}$$

**Fig. 27:**
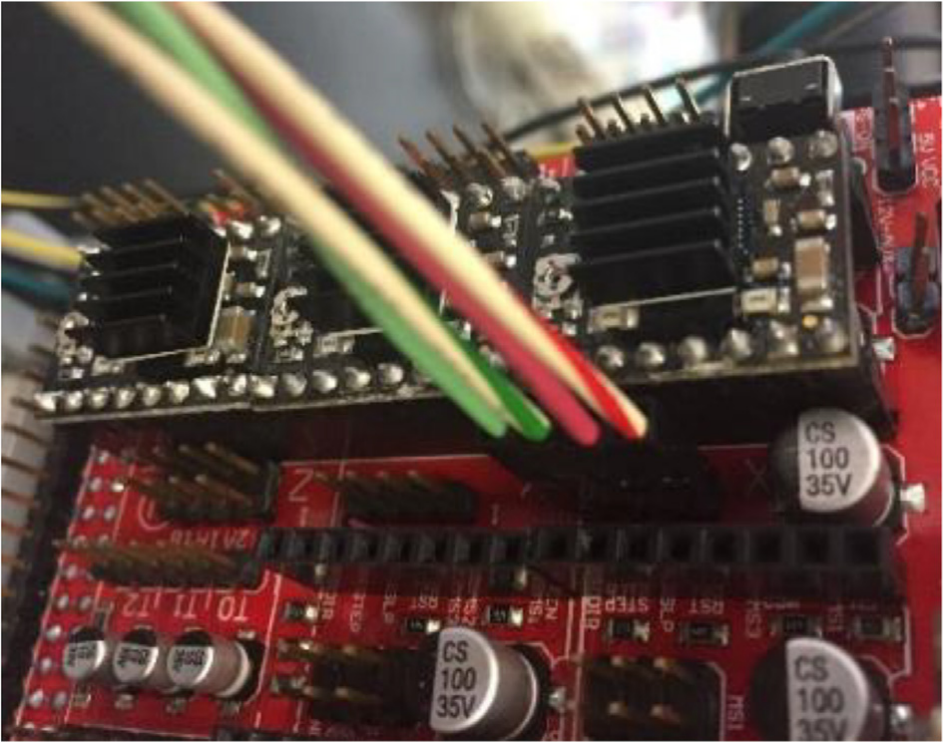
Connection of NEMA-17 motor wires to RAMPS board.

In this design, the datasheet indicated an *I_max_* value of 1.2 A, indicating a *V_ref_* of 0.65 V. To set *V_ref_*, tune the potentiometer on the bottom of the motor driver and monitor its voltage using a multimeter. An in-depth guide on this process can be found in Ref. [31]. Once this process is complete for all three motor drivers, connect the four wires from each NEMA-17 motor to the appropriate 1A, 1B, 2A, and 2B pins for the X-, Y-, and Z-axis motor drivers on the RAMPS board (Fig. 27).

5. Within the Raspbian OS of the Raspberry Pi, download the ‘ARMv6hf’ version of *Processing* from their website [32] and install. Then, download and extract the ‘*RAMPS.zip*’ from the Supplementary Information. Double click on any file within the extracted folder to open the user interface. Click the ‘Play’ button in the top left corner to initiate control of the system using the software.

### 5.2 Build instructions for SCARA system

#### 5.2.1 Base for SCARA system

1. The first major piece of the SCARA autosampler to be constructed is the base. The three required 14″ × 9″ base pieces (*Base_1.dxf*, *Base_2.dxf*, *Base_3.dxf*) can be laser cut from a single sheet of ¼” thick plywood (PN# 958719), as shown in Fig. 28. The dimensions do not need to be exact, though they should be at minimum 14″ × 9″. The plywood also does not need to be perfectly flat. Various plastics can also be used for a similar purpose, although some of the positioning pins and binding between the layers may need to be modified. Any warp in the board will be corrected during the gluing process. One of these sheets is to be laser cut as the very bottom layer of the base. The other two sheets are to be laser cut as the middle and top layers of the base, which are identical. After laser cutting the boards, the edges may be rough or have splinters. It may help to sand down the edges prior to continuing, though it is not necessary.

**Fig. 28:**
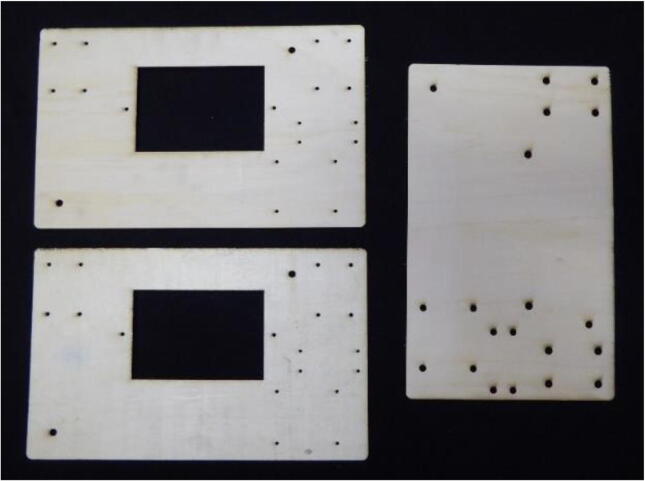
Three boards used to construct the base. These are laser cut from blank sheets of plywood.
2. Place down one of the scrap board pieces (PN# 914827, similar in dimension to the three base pieces) on a flat, level surface. Place a layer of paper towel over the scrap board to avoid adhesion from glue leakage Place the bottom base piece (the one not containing an open square in the middle) on top of paper towel, with the corner containing a single hole placed at the bottom left. Cover the top surface with wood glue (PN# 107209) and spread it around with a paper towel or brush: the goal is to have a relatively thick, even layer of glue spread out over the top surface, except for the region for the cutout hole on the top two base pieces (Fig. 29). Place the two dowel pins (PN# 98381A539) at the positions shown in Fig. 29, with the bottom of the dowel pin set to be level with the bottom of the bottom base piece.

**Fig. 29:**
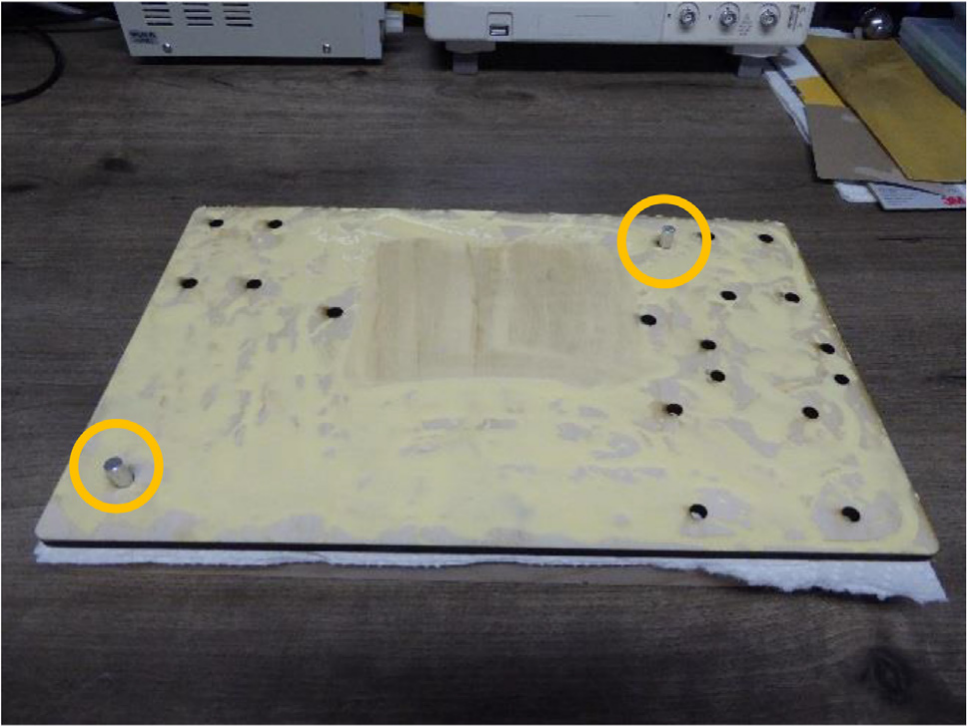
Bottom base piece with glue layer, placed on top of scrap board support piece. The yellow circle regions indicate the positions of the alignment dowel pins. (For interpretation of the references to color in this figure legend, the reader is referred to the web version of this article.)
3. Place one of the two remaining boards on top of the glue layer, using the dowel pins for alignment. Press tightly down, cleaning up any glue that is squeezed out along the edges or into the well plate hole in the middle of the base. On top of this new piece, place another layer of glue, spread it out evenly, and place the final base piece board on top (again using the dowel pins for alignment). Repeat the glue cleaning procedure along the edges and in the central recess. The full assembly is shown in Fig. 30.

**Fig. 30:**
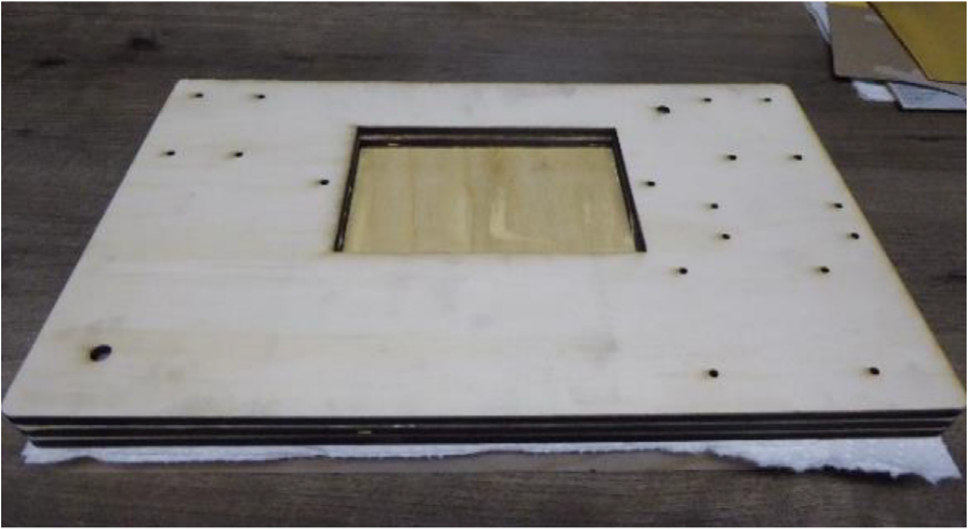
Three-layer base for SCARA autosampler design following glue distribution.
4. Place another paper towel on top of the base, followed by the remaining scrap piece of wood. Tighten 6–8 clamps around the stack (Fig. 31), with the scrap boards helping distribute the force and prevent indentations into the actual base. Wipe away any excess glue on the edges once the clamps are tightened.

**Fig. 31:**
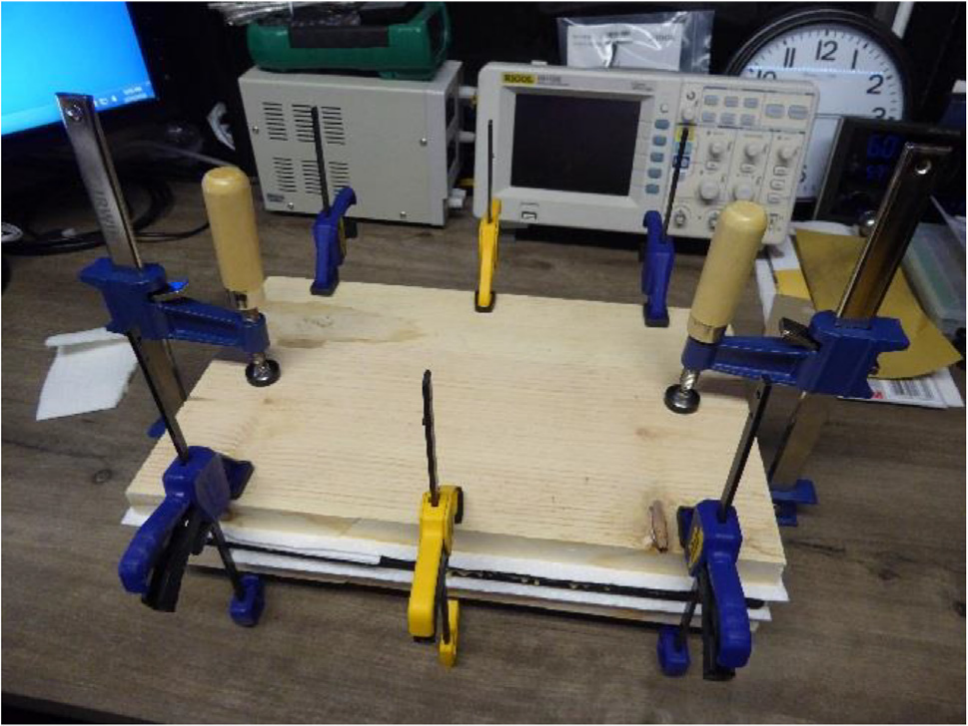
Tightened clamps used to hold the base stack together for glue curing.
5. Begin the glue curing process, which may take up to 24 h depending on the selected adhesive. To clean excess glue from the base once curing is complete, a razor (or similar) blade or sandpaper can be used to remove glue that has been squeezed out of the edges. A drill (#34 bit or smaller) or other cutting tool can be used to remove excess glue from the holes that did not contain the dowel pins. A completed SCARA base after curing and cleaning is shown in Fig. 32.

**Fig. 32:**
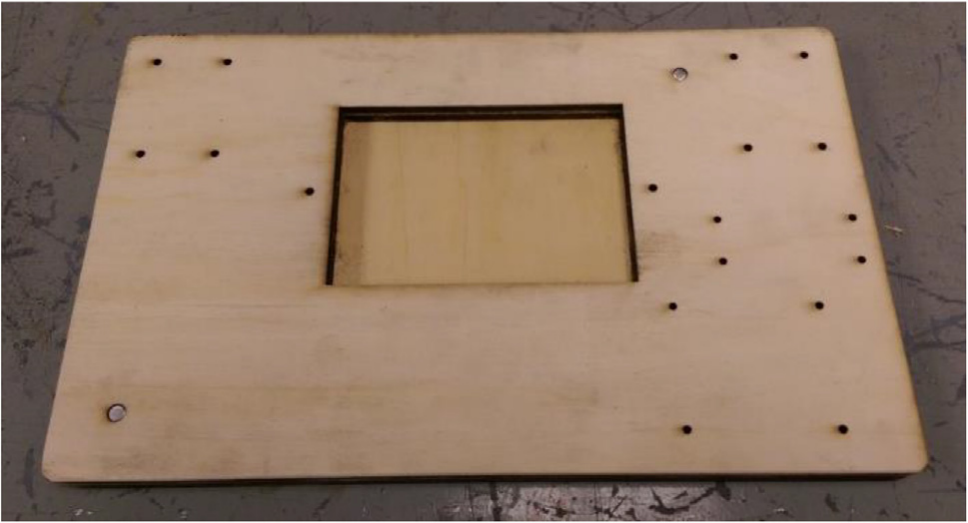
Completed assembly of the SCARA base.

#### 5.2.2 Servomotors for SCARA system

1. To assemble the left and right servomotor plates, insert four #4 × 0.75″ pan head screws into the four corners of each top piece (*ParallelMountA_top.STL* and *ParallelMountB_top.STL*, respectively), with the head of the screw placed into the recessed countersink hole so that it below the top surface of each plate top. Fit each plate front (*ParallelMountA_front.STL* and *ParallelMountB_front.STL*) and back (*ParallelMountA_back.STL* and *ParallelMountB_back.STL*) onto the screws: the thinner piece for the right and left servomotor modules should be on the side closer to the large hole cutout on each of the top plates. The rounded corners for all pieces will match when properly aligned. The slot cutout between the two holes for each front and back piece should be facing the center. Once the components are in the correct position, attach them by tightening a #4 nut onto each screw. The completed assemblies are shown in Fig. 33.

**Fig. 33:**
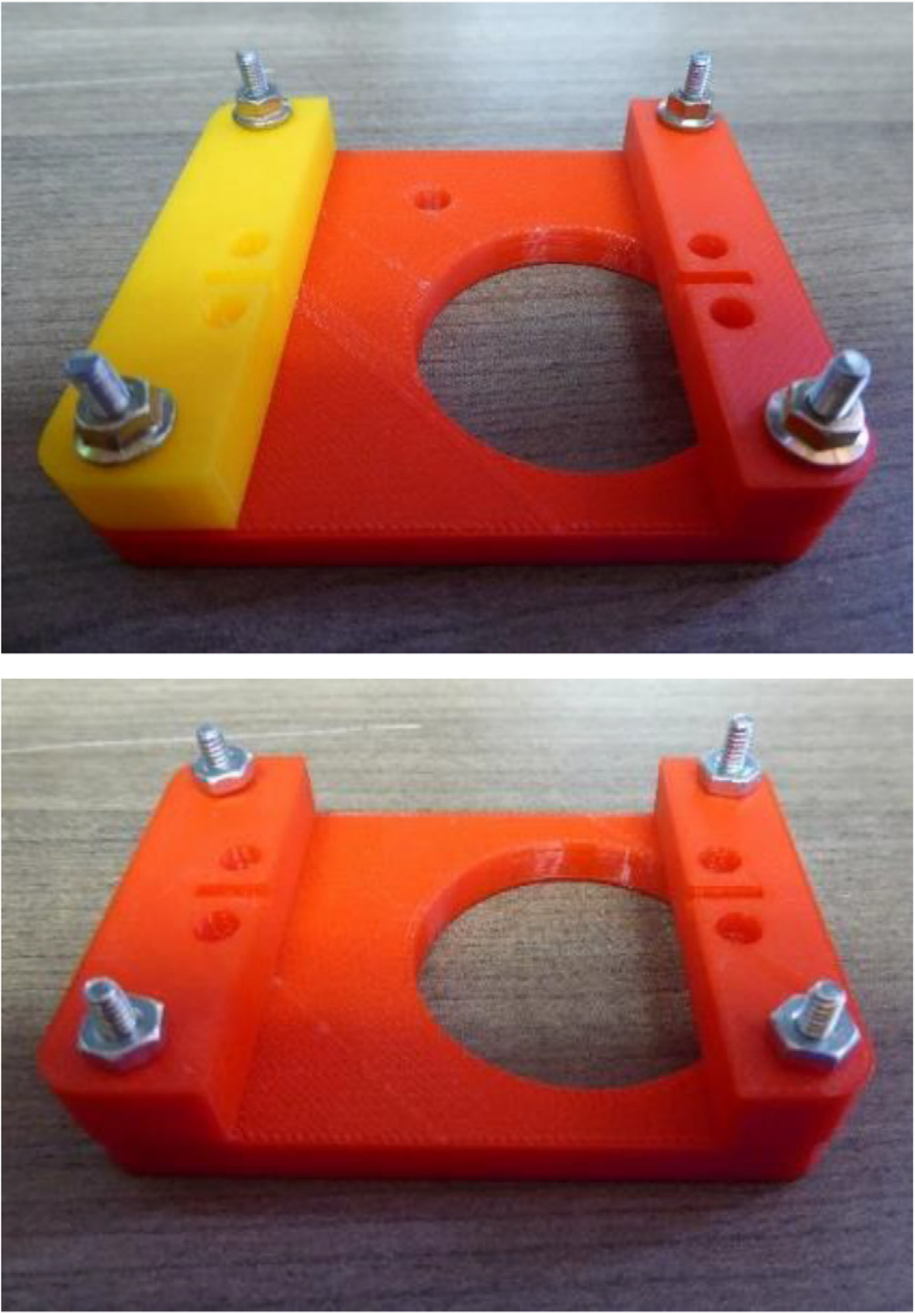
Completed left servomotor plate (top) and right servomotor plate (bottom).
2. To attach the left servomotor, place two M3 × 20 mm screws through the two holes on the top left corner of the base piece. The screw heads should be placed in the countersinks on the bottom of the base, with the threads coming up through the top. Place a servomotor stand (PN# 1804–0032-0001) on top of the threads and tighten in place using two M3 nuts (Fig. 34).

**Fig. 34:**
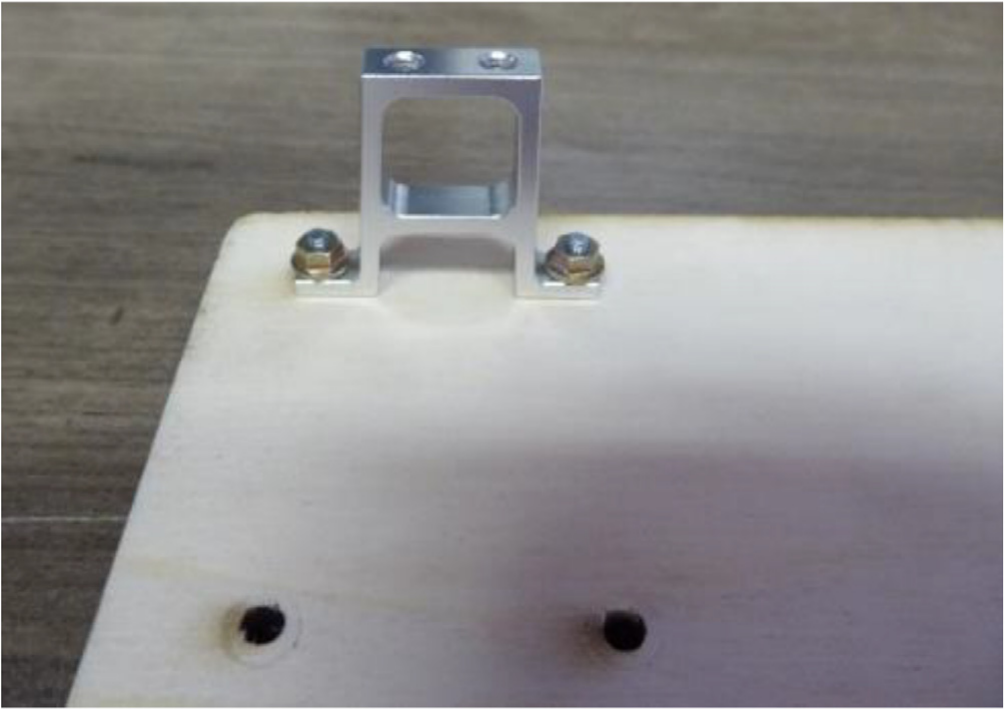
First servomotor stand attached for left servomotor apparatus.
3. Place two more M3 × 20 mm screws through the holes shown on the bottom of Fig. 34 with the threads facing up. Place the back (non-wired) side of a HS422 servomotor (PN# 31422S00) in place on the existing stand, then slide the front servomotor stand onto the screws, ensuring that the wires of the servomotor are below the stand. Tighten the stand in place using M3 nuts (Fig. 35).

**Fig. 35:**
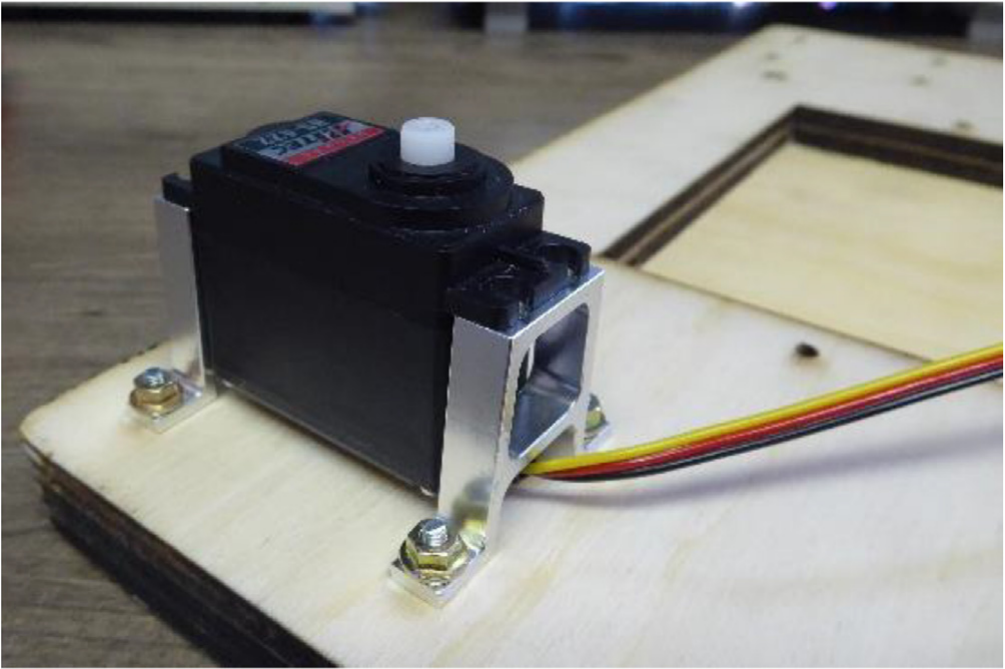
Left servomotor in position attached to two stands.
4. Align the combined left servomotor plate on top of the servomotor by placing the middle of the large hole over the servomotor spline and the four mounting holes on both the plate and motor/stand. Attach the plate to the motor and stand using four M4 × 20 mm screws tightened into the threaded holes on the stand. The completed component is shown in Fig. 36.

**Fig. 36:**
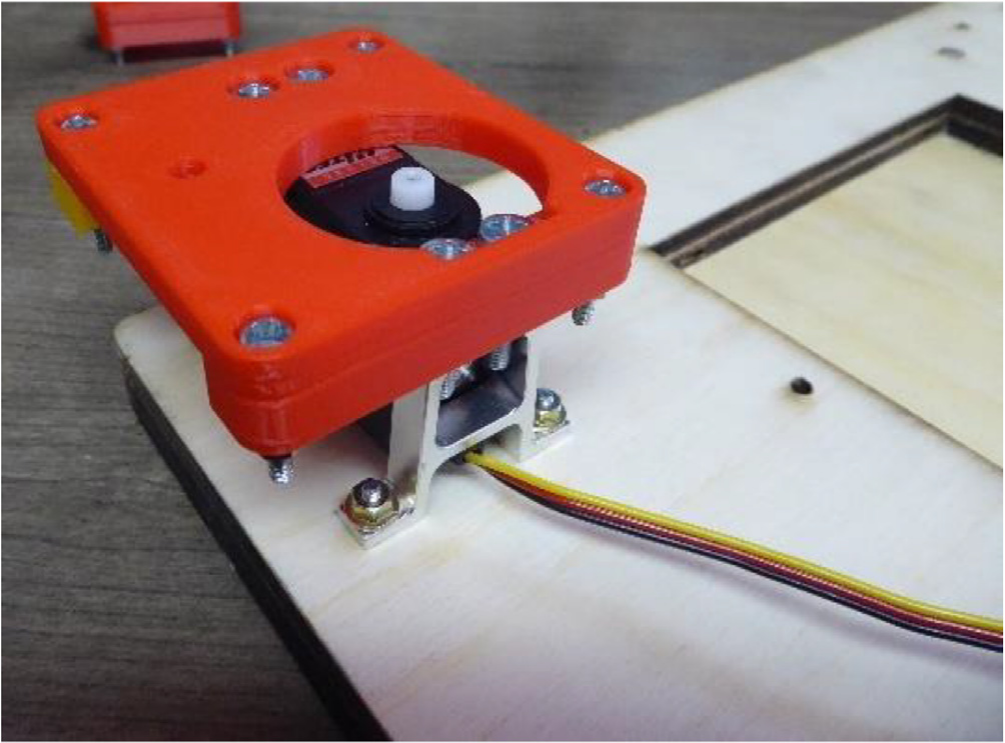
Combined servomotor and plate module on the left side of base.
5. The process for the attaching the right servomotor and plate is very similar, with only one small difference: M3 × 30 mm screws are used through the base, and the servomotor stands are designed to sit on the 3D printed servomotor stand riser (*Riser.STL*) rather than directly onto the base (Fig. 37). The completed right servomotor module is shown in Fig. 38 and the complete base with both servomotors is shown in Fig. 39.

**Fig. 37:**
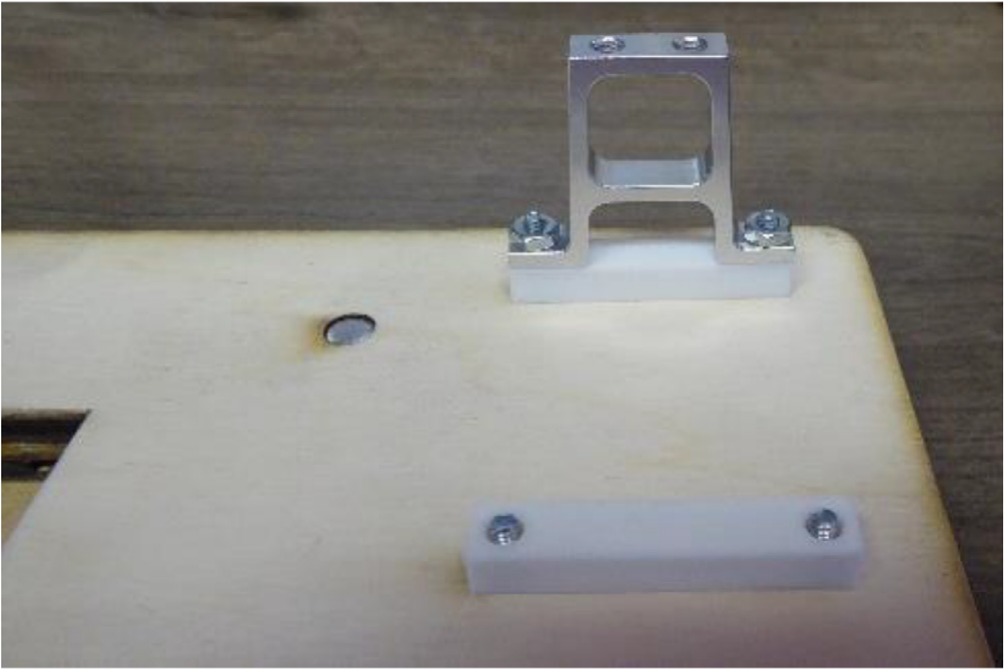
Use of servomotor stand risers on right servomotor stands.

**Fig. 38:**
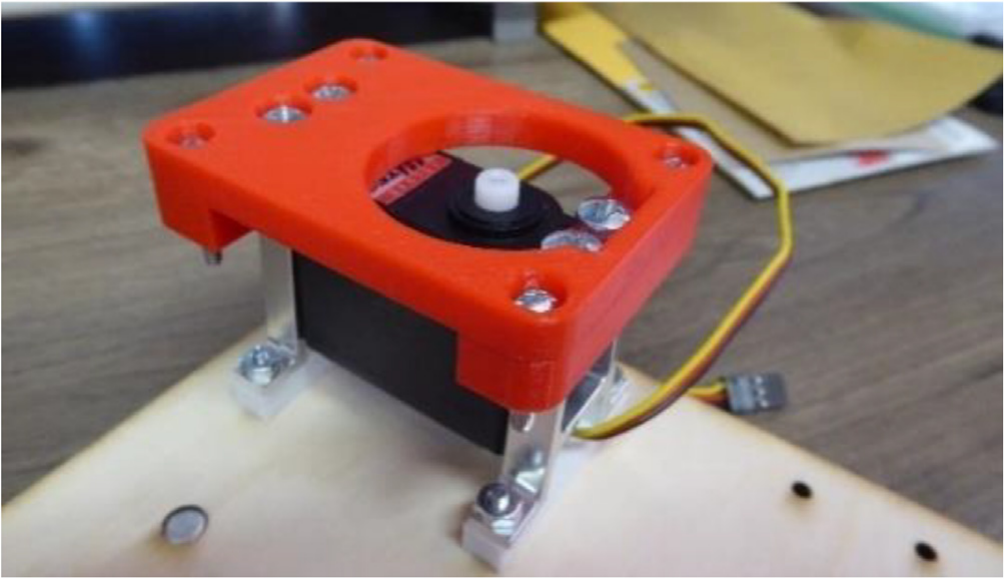
Combined servomotor and plate module on the right side of base.

**Fig. 39:**
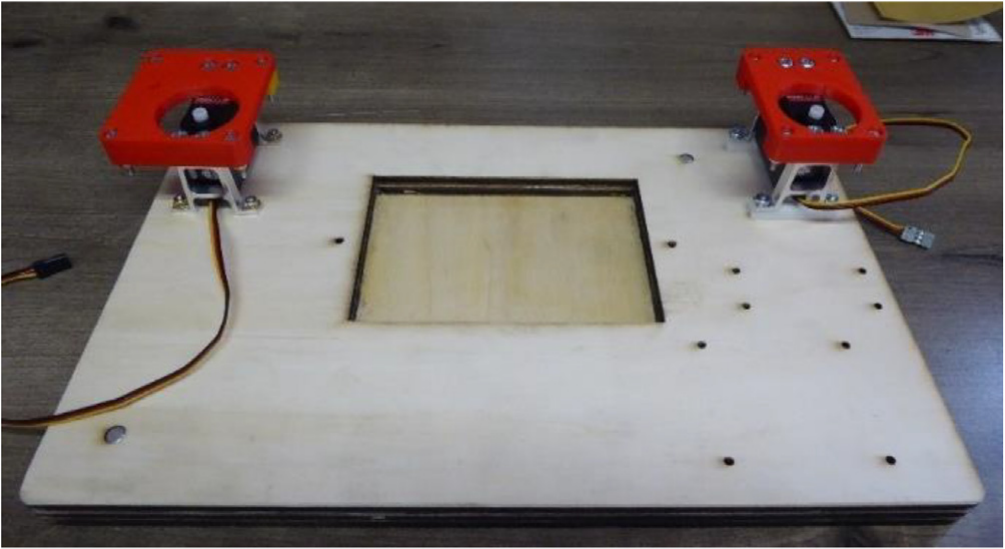
Completed assembly of the base with servomotors attached.

#### 5.2.3 Linkage for SCARA system

1. For the purposes of a sampler used for the generation of segmented flow droplet streams from a well plate, costs can be decreased by limiting the Z-axis position of the sampling capillary to “in-well” and “above-well” positions. For the lower-cost SCARA approach, this is achieved using a solenoid coil rather than a complete stepper motor design as with the 3-Axis autosampler. The first step in construction of a component that achieves this movement is the attachment of the solenoid coil body (included in PN# 1144–1419-ND) and the end effector *(EndEffector.STL*). Two #4 × 0.5” screws are placed through the holes on the rectangular section that is orthogonal to the larger part of the plate. Align the screws with the threaded holes on the coil body, making sure that the plunger opening is facing the same direction as the top of the end effector (Fig. 40). Tighten the screws into the thread holes to attach the two pieces.

**Fig. 40:**
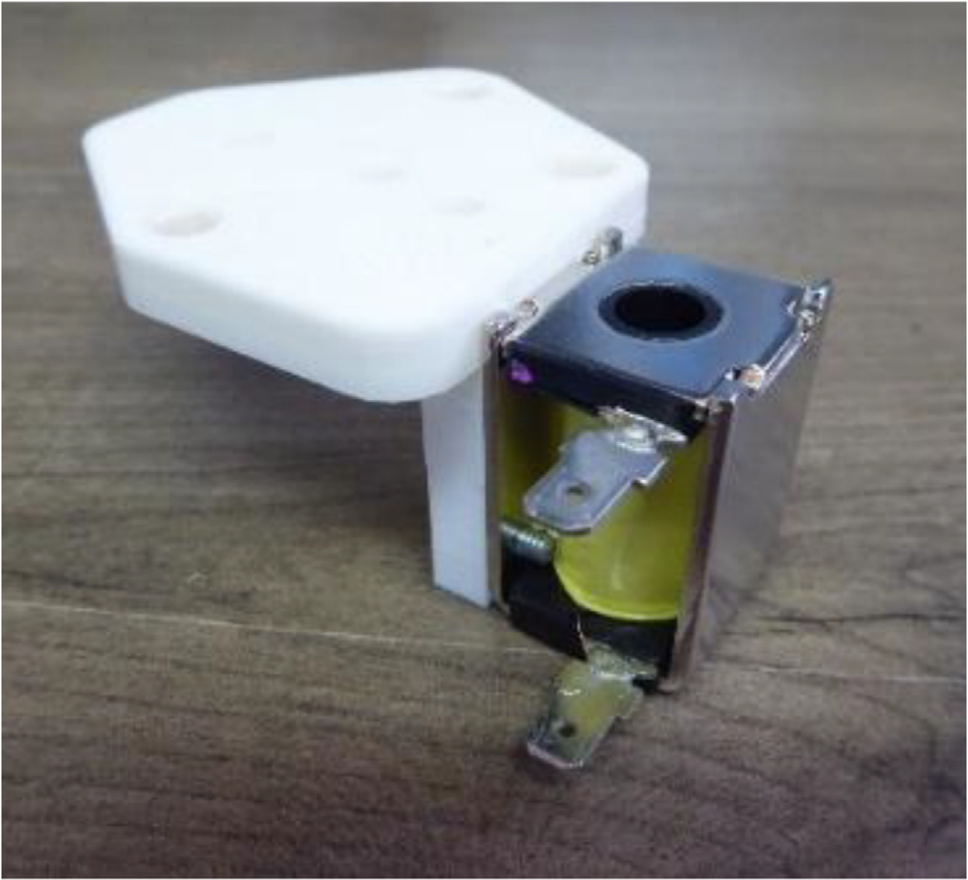
Connection of end effector to solenoid coil body.
2. Attach the capillary rail (*CapillaryRail.STL*) and lever fulcrum (*Fulcrum.STL*) onto the top of the capillary plate using #4 × 0.5″ screws and #4 nuts in the positions shown in Fig. 41.

**Fig. 41:**
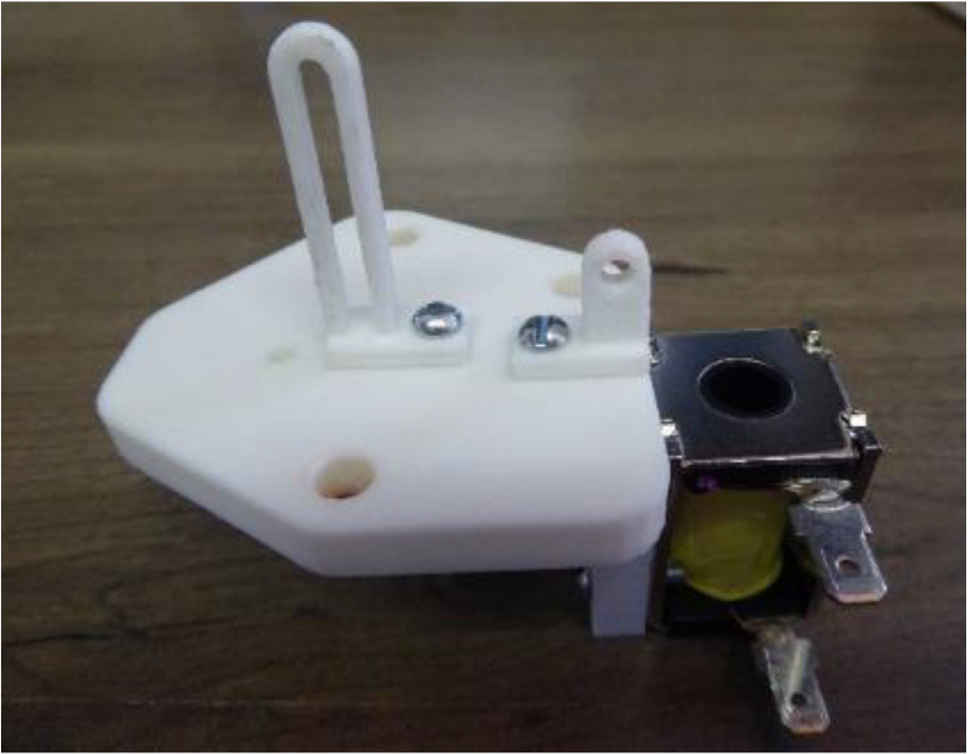
Capillary rail and lever pivot attached to top of capillary plate.
3. Arrange the lever attachment (*LeverAtt.STL*) and the main lever piece (*LeverMain.STL*) so that the longer holes have a gap between them. Connect the two pieces through the smaller holes using a #4 × 0.5″ screw and #4 nut. Then, attach the solenoid plunger to the other side of the main lever piece using a M2 × 10 mm screw and M2 nut (Fig. 42). One hole at the center of the combined piece should remain open for connection in the next step.

**Fig. 42:**
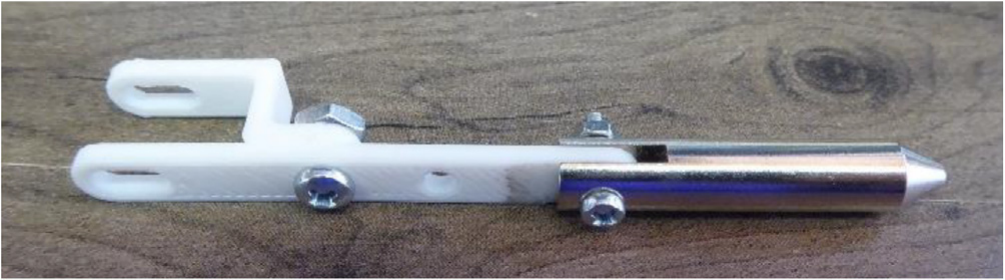
Combined lever and plunger assembly.
4. To begin attaching the lever-plunger assembly to the capillary plate, cut the compression spring (PN# 9657 K107) to a length of approximately 0.75″, slide it over the plunger, and insert the pointed end of the plunger down into the solenoid coil body. Align the central lever hole with the lever pivot hole and connect with a #4 × 0.5″ screw. Place a drop of threadlocker (PN# 1810A27) onto the exposed threads of this screw, hand-thread the nut onto the screw, and let the threadlocker cure for 10 min before continuing. Do not fully tighten the nut, as that will restrict the motion of the lever. Attach the capillary clamps (*CapillaryClampA.STL* and *CapillaryClampB.STL*) with #4 × 0.75″ screws so that they ride within the capillary rail. The capillary guide (*CapillaryGuide.STL*) is then attached to the bottom of the capillary plate with #4 × 0.75″ screws. The complete assembly is shown in Fig. 43. Attach the solenoid to a 12 V power supply and toggle the power. If the solenoid is able to fully retract the spring, then the spring length is acceptable. Otherwise, the spring should be cut further. It is recommended not to cut more than half a winding at a time.

**Fig. 43:**
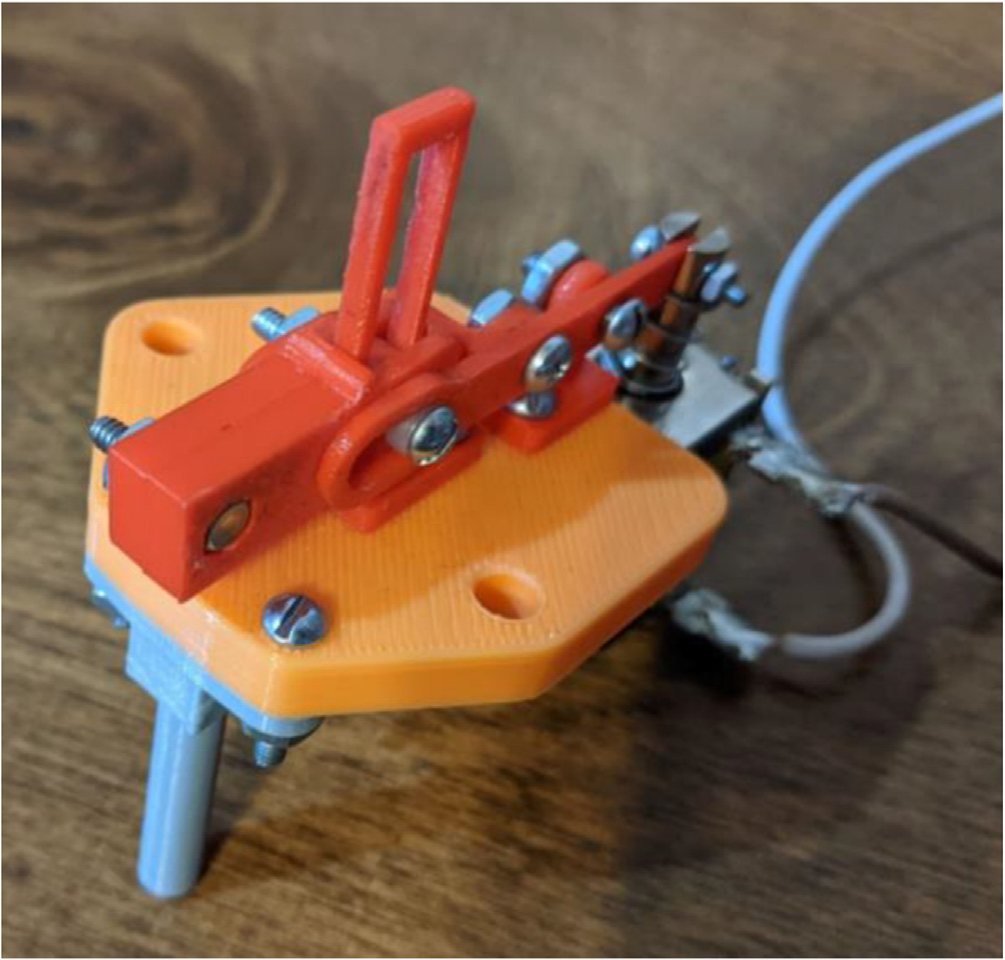
Connection of capillary plate, solenoid with plunger, and lever.
5. To begin preparing the linkages that are connected to the capillary plate, attach the R-I (Regular, I-shaped) horns onto links A1 (*LinkA1.STL*) and B1 (*LinkB1.STL*), with the spline hole in the horn aligned with the larger hole on the link and the wings of the horn in line with the slots. Tighten two #0 × 0.5″ thread-forming screws through the link and into the horn so that the threads are on the same side as the horn. The screw for the inner slot can be placed in any of the holes of the horn wing. The screw for the outer slot should be placed in the hole closest to the spline. One of the horn-link connections is shown in Fig. 44. Set aside link A1 and attach links B1 and B2 (*LinkB2.STL*) using a 0.2″ × 0.5″ Chicago screw, adding threadlocker to the threads before it is tightened. Let the connected pieces sit until the threadlocker sits. (*Note*: *use a similar threadlocker process for all subsequent Chicago screw connections*).

**Fig. 44:**
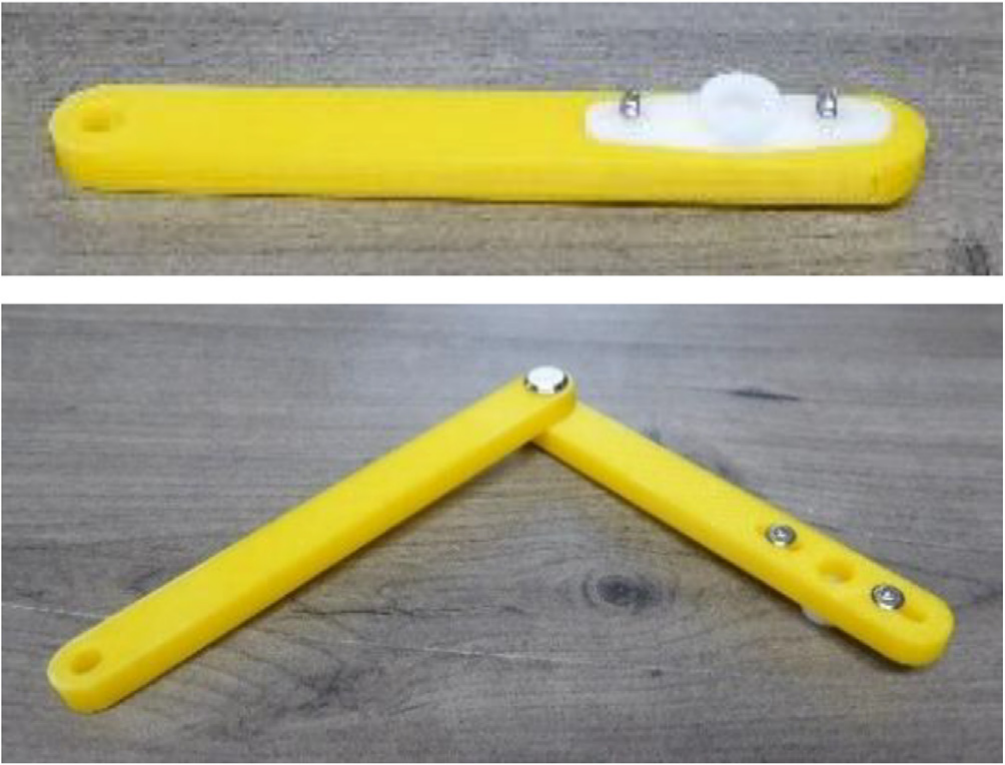
Link B1 connected to the R-I horn (top) and connection between links B1 and B2 (bottom).

**Fig. 45:**
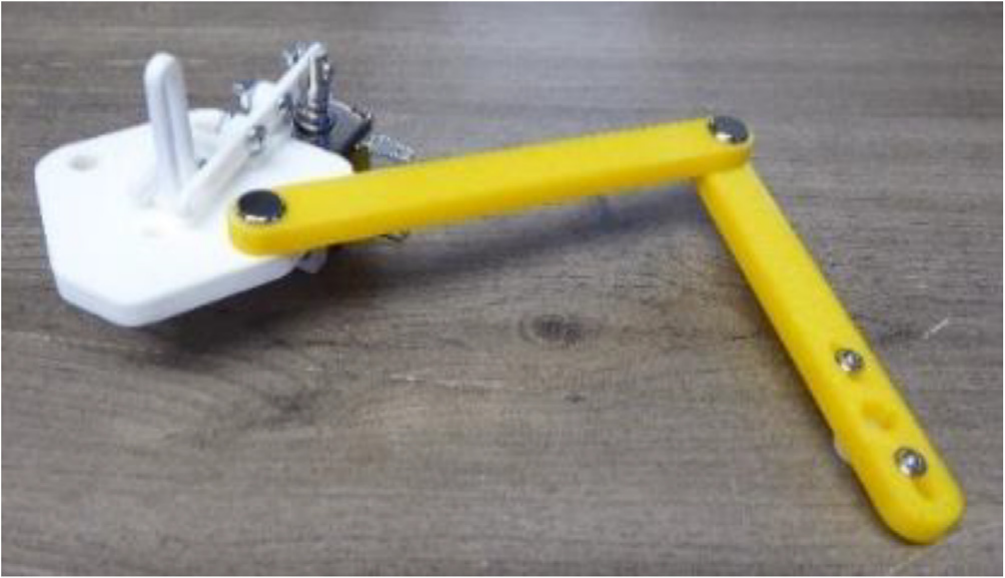
Connection of capillary plate and right sub-linkage.
6. Attach the combined B1/B2 link (right sub-linkage) to the hole on the right side of the capillary plate with a 0.2″ × 0.5″ Chicago screw (Fig. 45).
7. Attach links P1 (*LinkP1.STL*) and P2 (*LinkP2.STL*) to the elbow (*Elbow.STL*). It is important that the elbow be oriented correctly since it is not symmetric. With the longest flat edge up, the shortest flat edge should be on the right (Fig. 46). With the elbow in this orientation, link P1 should be aligned so it is concentric with the left hole of the elbow and is under the elbow, and link P2 should be aligned so it is concentric with the right hole of the elbow and is on top of the elbow. Both are attached with 0.2″ × 0.5″ Chicago screws.

**Fig. 46:**
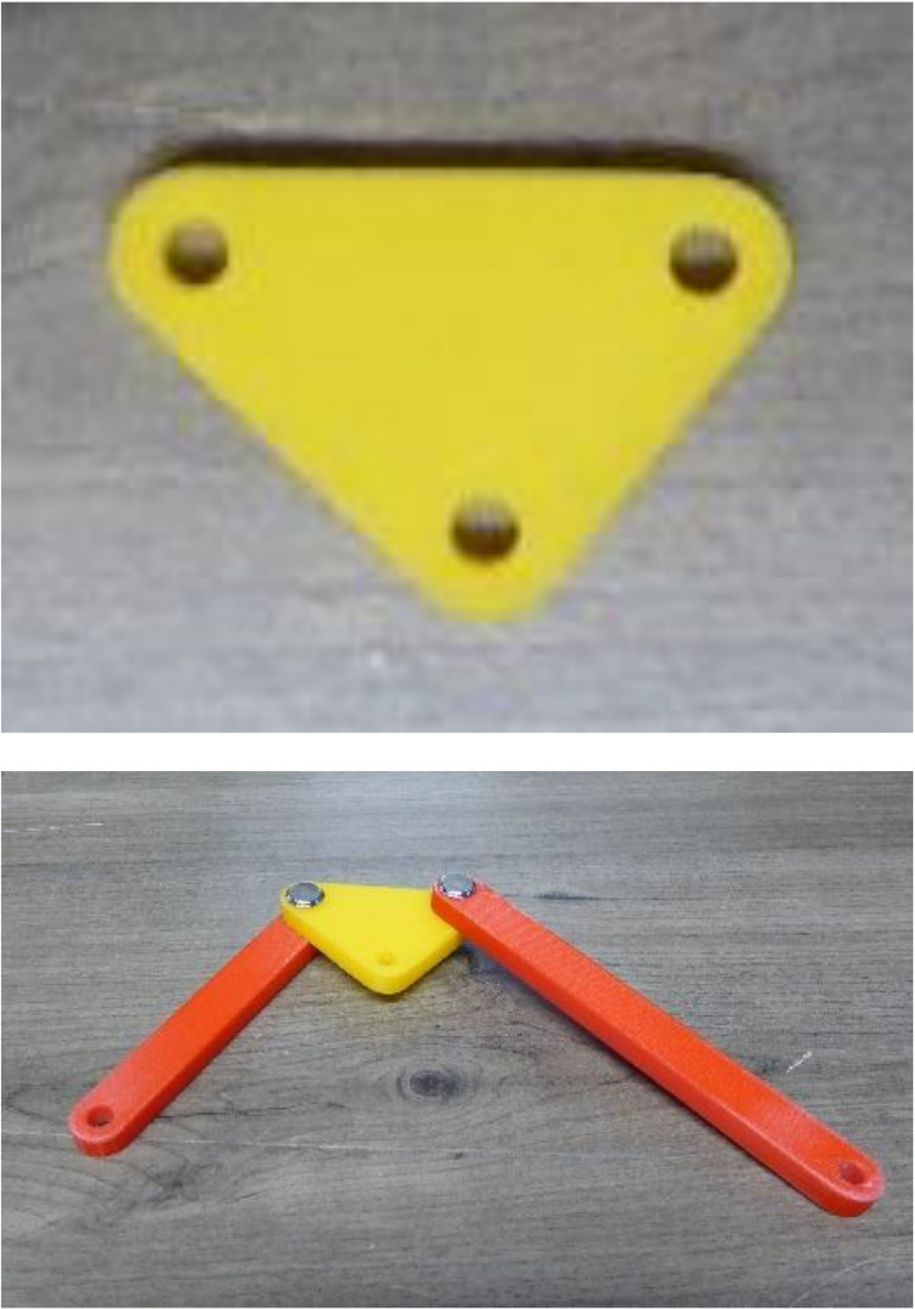
Orientation of elbow piece for linker connection as described in Step 7 of *Section 5.2.3* (top) and connection of elbow piece with links P1 and P2 (bottom).
8. Connect links A1 and A2 (*LinkA2.STL*) to the remaining hole on the elbow (Fig. 47). Link A1 should be aligned under the elbow (with the horn positioned away from the elbow and pointing down) and link A2 should be aligned on top of the elbow, with all three pieces connected using a 0.2″ × 0.6875″ Chicago screw.

**Fig. 47:**
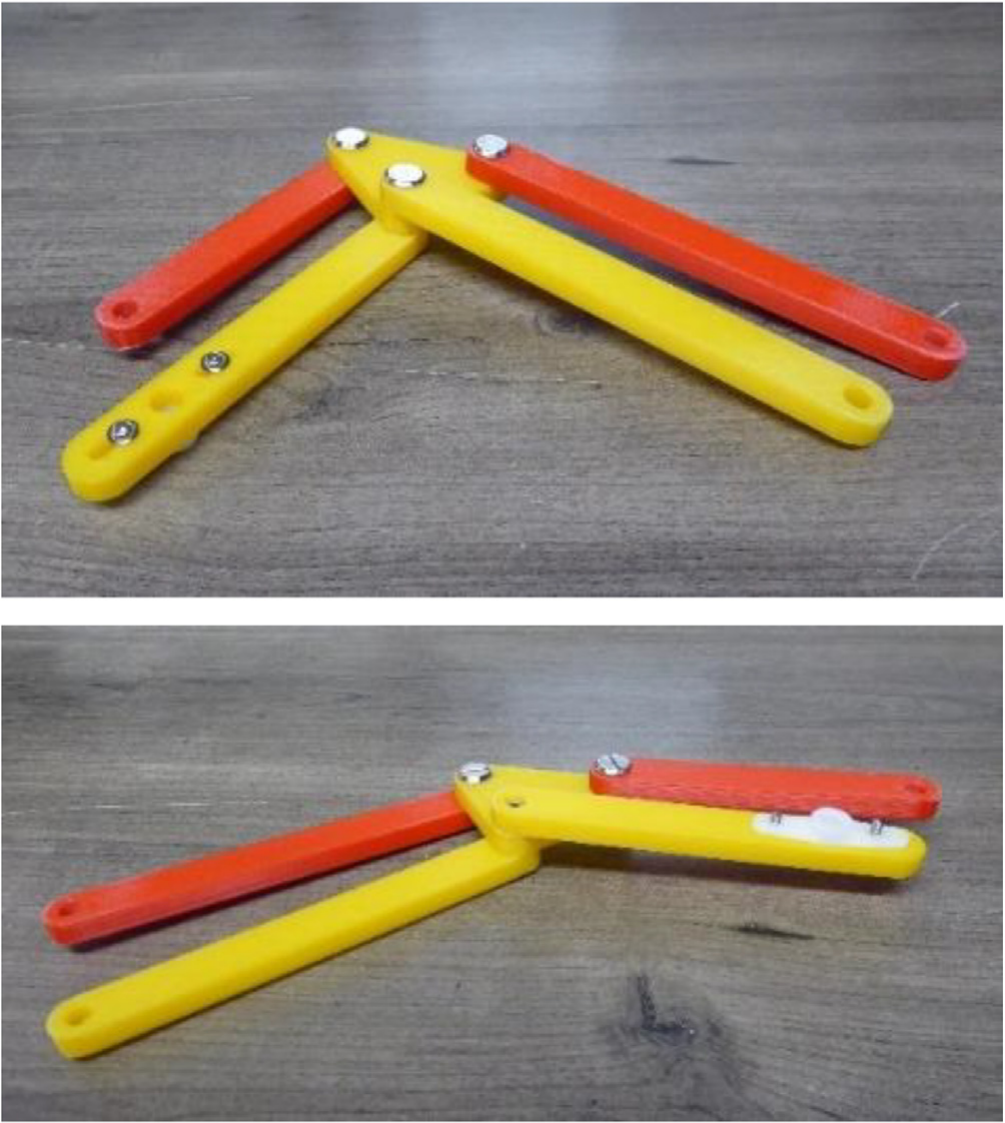
Top view (top) and underneath view (bottom) of the elbow connected to links A1, A2, P1, and P2 (complete left sub-linkage).
9. Attach the completed left sub-linkage to the capillary plate as shown in Fig. 48 using 0.2″ × 0.5″ Chicago screws.

**Fig. 48:**
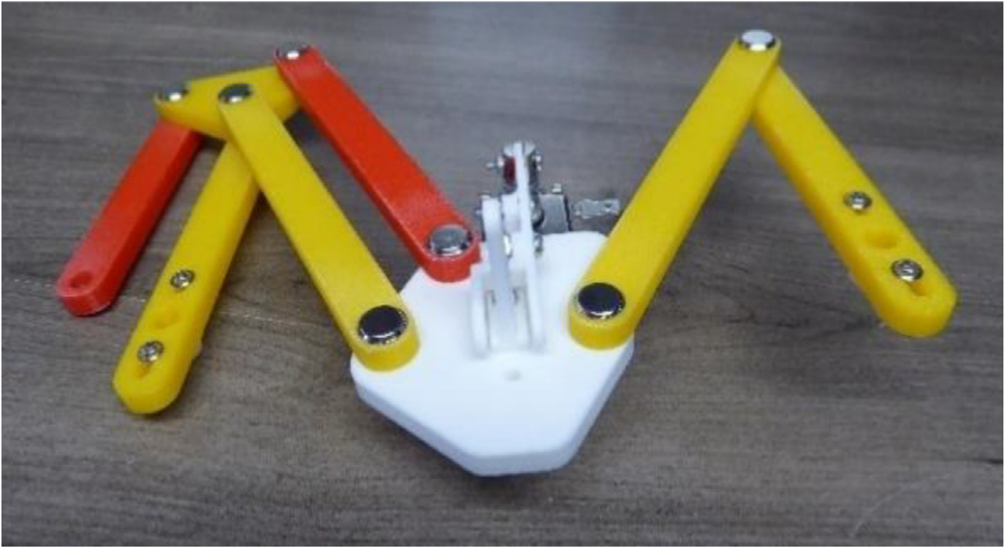
Connection of left and right sub-linkages to capillary plate.
10. Align the horn on the right sub-linkage (link B1) with the spline on the right servomotor and tighten with a spline screw (Fig. 49).

**Fig. 49:**
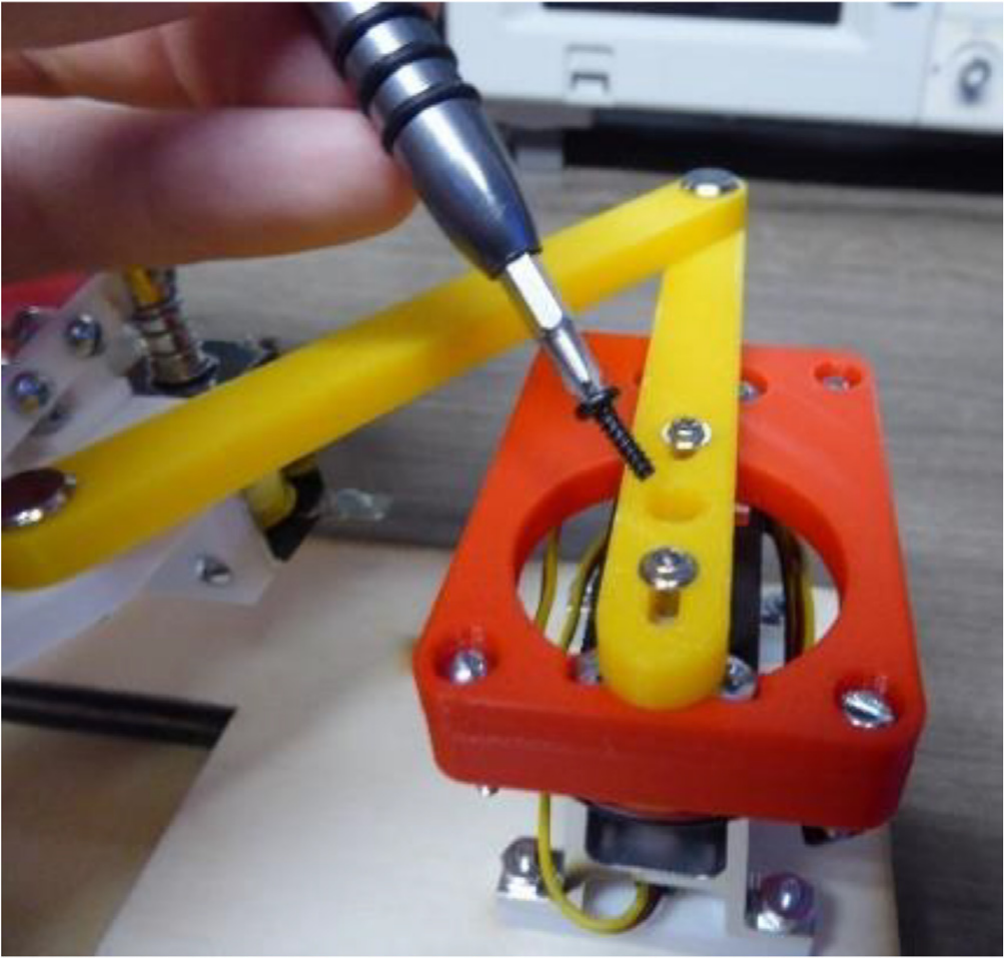
Connection of right sub-linkage to right servomotor with spline screw.
11. Complete the linkage assembly by aligning the horn on the left sub-linkage (link A1) with the spline on the left servomotor and tightening with a spline screw. Then, connect the remaining hole on link P1 to the remaining hole on the left servomotor plate with a 0.2″ × 0.5″ Chicago screw. The completed linkage assembly is shown in Fig. 50.

**Fig. 50:**
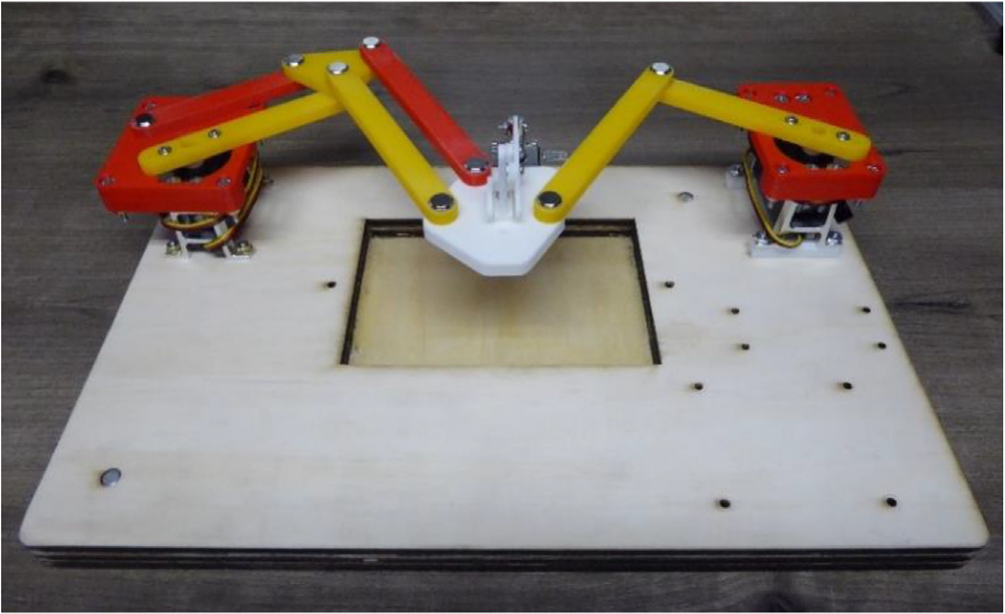
Completed linkage assembly connecting capillary plate and two servomotors.

#### 5.2.4 Raspberry Pi platform on SCARA frame (Optional)

**Fig. 51:**
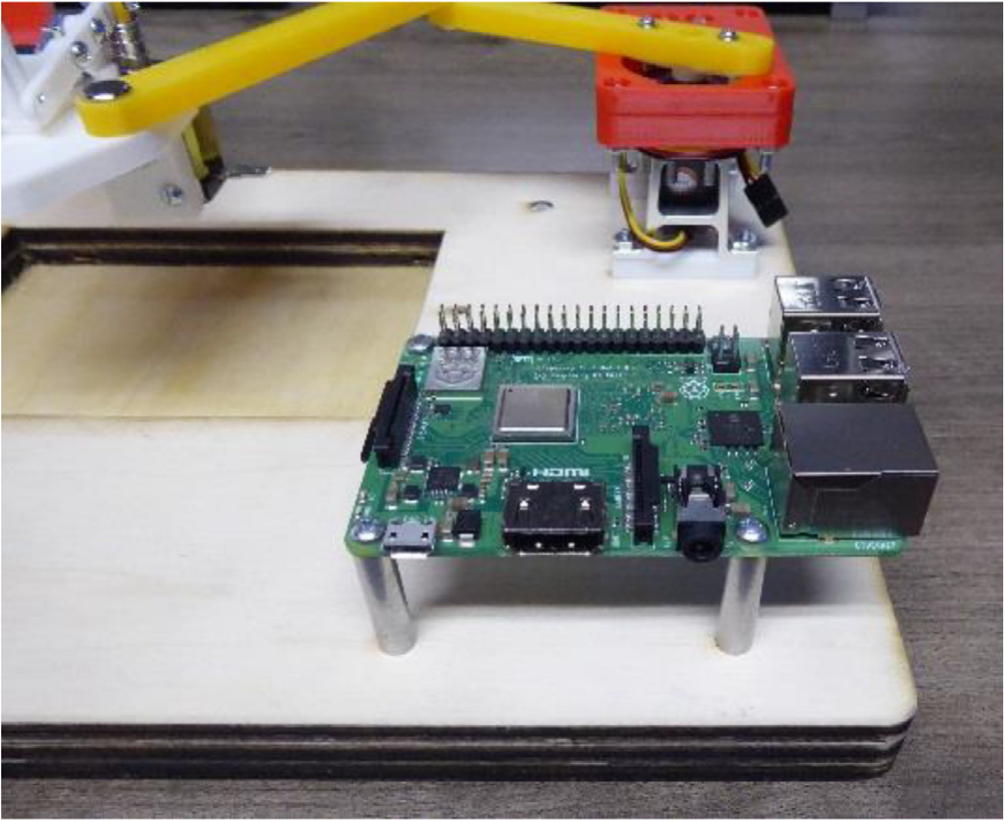
Raspberry Pi attached to SCARA base.

Although the Raspberry Pi does not need to be connected to the base, four holes are open in the bottom right hand corner of the base. Four M2.5 × 15 mm screws can be oriented with the threads facing up through the bottom of the base and connected to four M2.5 × 10 mm standoffs. Once the standoffs are in place, position the four corners of the Raspberry Pi onto the standoffs and connect with M2.5 × 5 mm screws. The finished position of the Pi is shown in Fig. 51.

#### 5.2.5 Raspberry Pi connections and software Installation for SCARA autosampler

Detailed instructions for soldering the components for the power supply and control boards are included in the ***Supporting Information***. The following steps can be performed once the boards have been completed.

1. Plug the power supply board into the control board, as shown in Fig. 52, making sure that the Raspberry Pi header and power supply header are both connected securely. Connect the 12VDC power supply (PN# Q1185-ND) to the power jack and plug it into an outlet. The three LEDs should light up when the power switch is turned on.

**Fig. 52:**
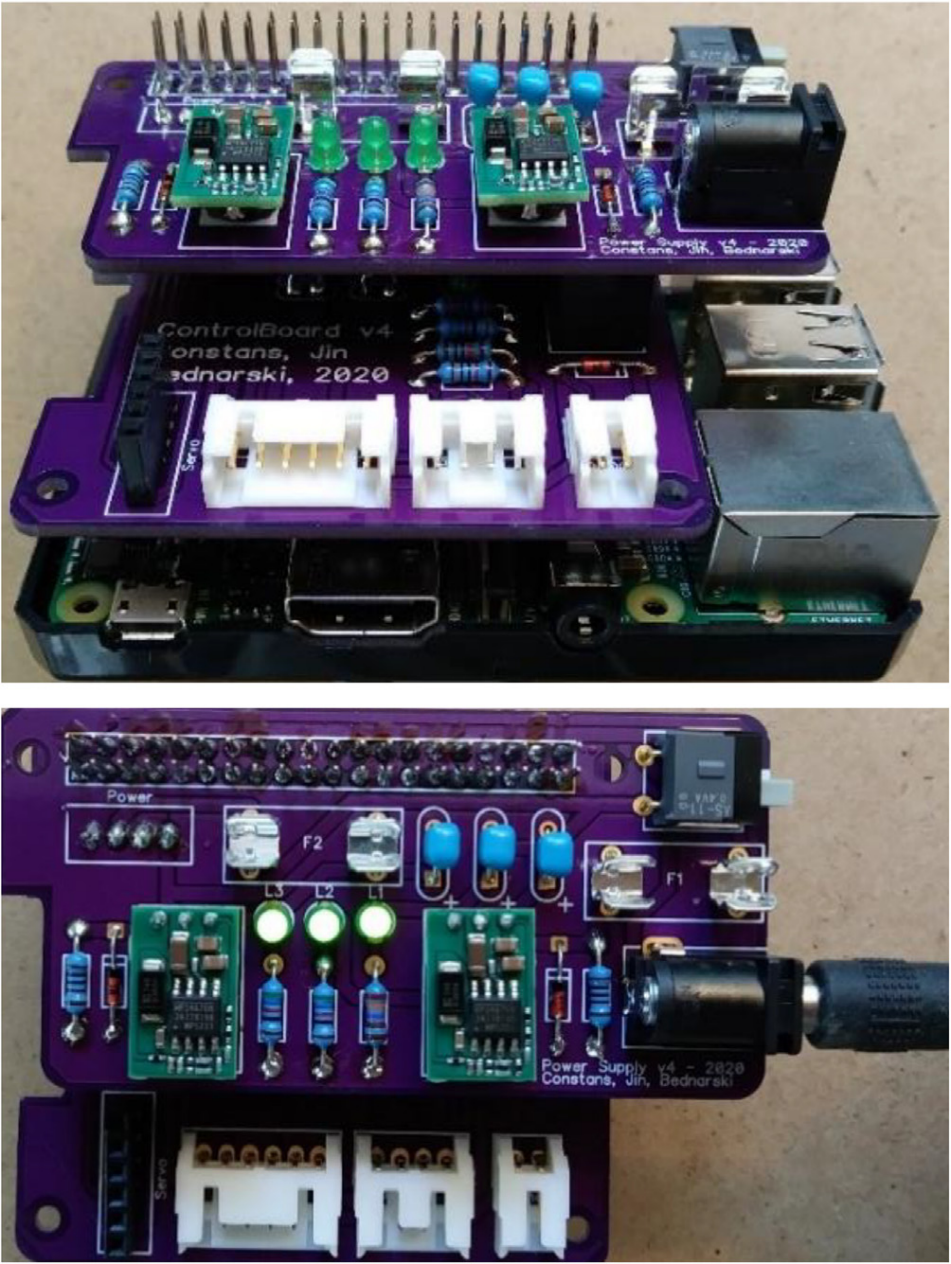
Stack of Raspberry Pi, control board, and power supply board (top). Three LEDs light up when power supply board is plugged in and power switch is turned on (bottom).
2. Plug the wires for the joystick and buttons into the joystick board and control board as shown in Fig. 53.

**Fig. 53:**
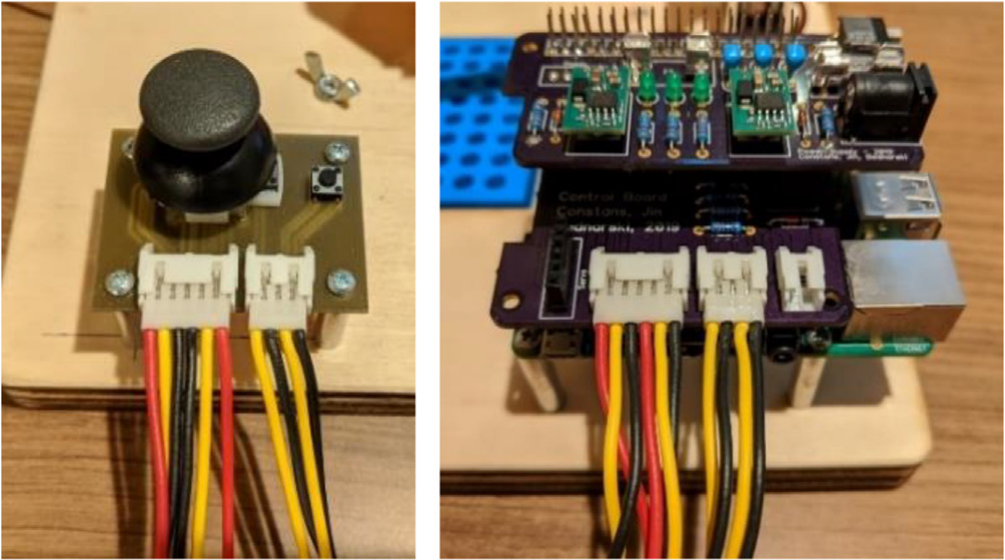
Connections between the joystick and the control board.
3. Solder the ends of the solenoid wire to the solenoid and then connect the solenoid to the header on the control board as shown in Fig. 54.

**Fig. 54:**
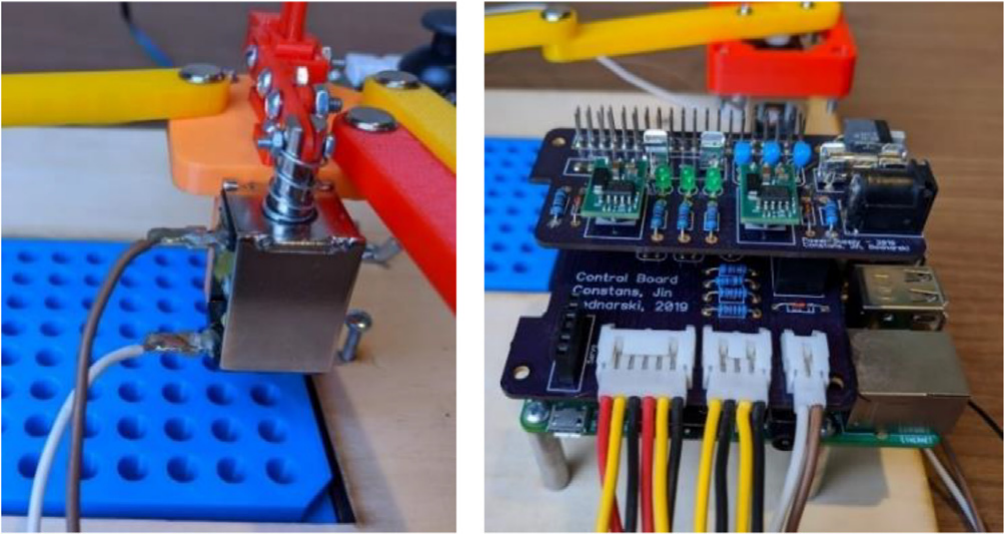
Connections between the solenoid and the control board.
4. Attach the servo control board to the header on the control board as shown in Fig. 55. Connect the servo on the left of the board to ***Connector 0*** on the control board. The right servo should be connected to ***Connector 1***. Ensure that the signal (yellow or white) wire is facing up when connected (Fig. 55).

**Fig. 55:**
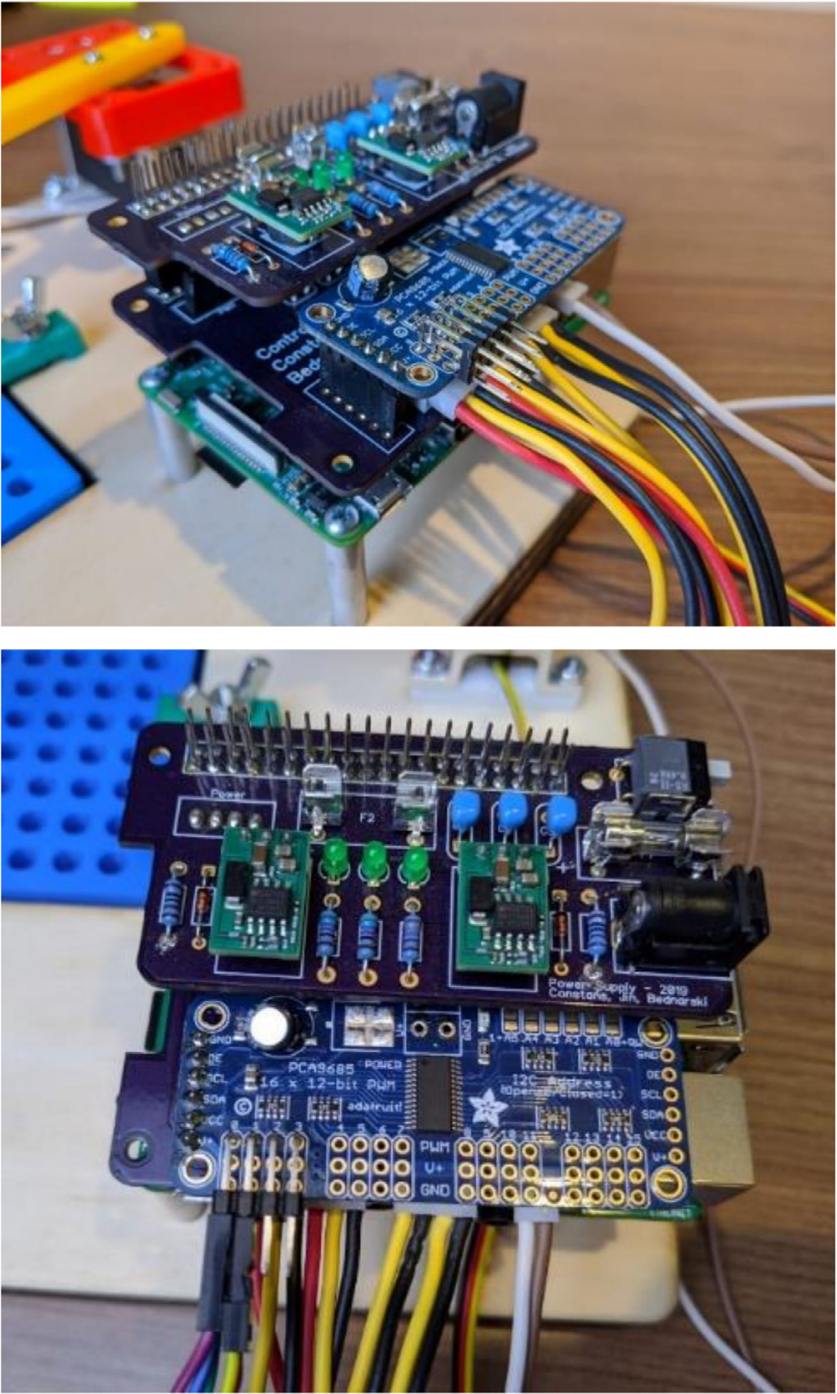
Connections between the solenoid and the control board.

**Fig. 56:**
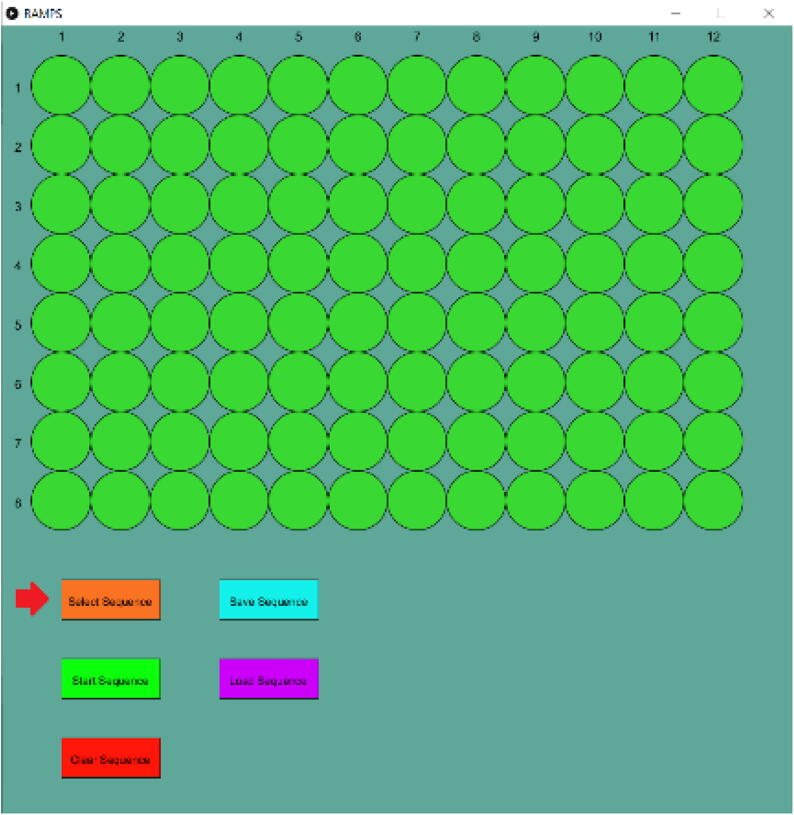
Graphical user interface for 3-Axis Autosampler.

**Fig. 57:**
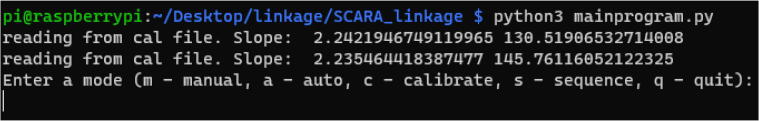
Command line interface for SCARA Autosampler.

## 6 Operation instructions

### 6.1 Operation instructions for the 3-axis autosampler

1. To generate movement between a consecutive sequence of wells, click the “Select Sequence” button (Fig. 56). The software will the record each well selected by the user. When a well is clicked, it will prompt the user to enter the length of time to remain in the well. After all wells in the sequence are selected, click the “Select Sequence” button again to stop recording.
2. Once a sequence has been selected or loaded, click the “Start Sequence” Button to begin the method. If no sequence is selected, nothing will happen.
3. If a wrong well is selected, or a sequence is done with use the “Clear Sequence” button can be clicked. It is important to note that this will ***completely*** clear any sequence currently loaded.
4. If you want to save a currently selected sequence to be imported later click the “Save Selection” button. This will pull up a prompt to select the file save location as well as the file name.
5. If you wish to load a saved sequence, click the “Load Sequence” button. This will bring up a prompt allowing the user to select a sequence file. The selected file will be loaded and can be started with the “Start Sequence” button. The loaded sequence can also be added to by clicking the “Select Sequence” button, but it should be noted that any additional wells selected will be added to the end of the loaded sequence.

### 6.2 Operation instructions for the SCARA autosampler

The SCARA Autosampler offers several modes accessed through a command line interface (Fig. 57). Run the python program *mainprogram.py*. The first time the program is run it will prompt the user to perform an initial calibration. Use the x axis of the joystick to control the rotation of the right servo and the y axis for the left servo. Move the sampler head to the vial indicated by the program and press in the joystick. The initial calibration is approximate so getting the sampler within about one centimeter is acceptable. It is recommended to rerun calibration mode after initial setup for more accurate results. After the final point, the program will create a folder in the working directory and save the calibration file. The program will then display the main menu. The program menu lists each of the possible operating modes. Access each mode by typing the corresponding letter for each mode and pressing enter.

1. **Manual Mode – “m”**: In manual mode, the program will ask for the row and column of the desired vial. Enter the row using the letter designator (A-H) and the column with the number (1–12). After pressing enter, the linkage will move to that position. The linkage will print out the x and y coordinates of the sampling head. Press the joystick down (button 1) to clear the entry and enter a new vial.
2. **Auto Mode** – **“a”**: This mode will step through all of the vials on the microplate. It starts at A1 and steps through all columns before moving to the next row. It starts at column 1 for each row.
3. **Sequence Mode – “s”**: The program will prompt for a sequence of vials to step through. The vials should be separated by commas in the format “A1, B2, C3, etc.” Press enter to start the sequence.
4. **Calibrate Mode – “c”**: This mode calibrates the autosampler by moving to a set of points and having the user align the sampling head using the joystick. It will overwrite the existing calibration files (./calibration/cal0.csv and ./calibration/cal1.csv) or create new files if they do not exist. The linkage will move near the first calibration point and ask the user to use the joystick. Move the sampling head directly over the vial and press the joystick (button 1) when aligned. Repeat this process for the remaining calibration points.
5. **Quit – “q”**: Exits the program.

## 7 Validation and characterization

### 7.1 Precision comparison of autosampler designs

To determine the motion characteristics of each movement design, a protocol based on ISO 9283 [22] was used as a guide in developing a comparison test. For the 3-axis autosampler, each axis was tested by moving to a central “home position”, then moving a distance *l* (1″ for the 3-axis system and 1.06″, equivalent to 3 well positions, for the SCARA system) and recording the new position with a dial caliper, and finally returning to the “home position”. Measurements for the 3-axis design were made with calipers that provide 0.0005” resolution (Mitutoyo America, Aurora, IL). This process was then repeated five more times, with accuracy calculated by:

(2) $$\mathrm{Accuracy}=\sqrt{{\left(\overset{-}{a}-l\right)}^{2}}$$

where *ā* is the mean value of the final calculated position for all trials for a given axis. The repeatability was then calculated using the following series of equations:

(3) $$d_{i}=\sqrt{{\left(a_{i}-\overset{-}{a}\right)}^{2}}$$

(4) $$\overset{-}{d}=\frac{1}{n}\sum_{1}^{n}d_{i}$$

(5) $$S_{d}=\sqrt{\frac{1}{n-1}\sum_{1}^{n}{\left(d_{i}-\overset{-}{d}\right)}^{2}}$$

(6) $$\mathrm{Repeatability}=\overset{-}{d}+3S_{d}$$

where *a_i_* is the deviation for an individual trial. Bi-directional movements were used for this test (a slight deviation from ISO 9283) to account for the potential of leadscrew backlash in the measurement. For the SCARA autosampler, the same process was conducted, although the Z-axis was not tested as it only moves in two positions based on the solenoid control. Measurements for the SCARA design were made with a dial indicator that provides 0.001″ resolution (Fowler High Precision, Newton, MA). Results of the process are shown in Table 8.

No issues were encountered during the development and routine operation of either platform described here, although no in-depth study on total performance lifetime was conducted. Due to the modular nature of these designs, any component that fails can be changed without the need to replace the entire system.

### 7.2 Use of 3-Axis autosampler for generated of segmented flow droplet stream

**Fig. 58:**
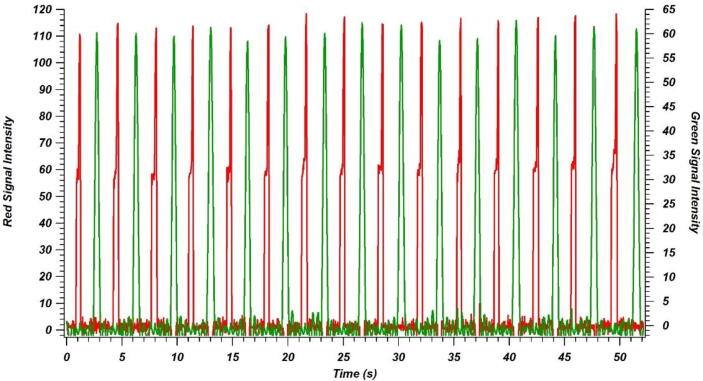
Intensity plot (RGB value) for portion of segmented flow droplet stream generated by alternating withdrawal between red and green food dyes in adjacent wells on a 96-well plate. Movement was controlled using the 3-axis autosampler. (For interpretation of the references to color in this figure legend, the reader is referred to the web version of this article.)

To demonstrate the use of the 3-axis autosampler for droplet formation, two adjacent wells of a 96-well plate were milled slightly below the planar surface, filled with green and red food dye (McCormick & Co., Inc., Hunt Valley, MD), respectively, and then covered with a layer of perfluorodecalin (PFD) oil (Alfa Aesar, Tewksbury, MA) up to the planar surface. A 44 cm segment of 100 µm inner diameter (i.d.), 360 µm outer diameter (o.d.) perfluoroalkoxy (PFA) tubing was coupled to a 250 µL gas-tight syringe (Hamilton, Franklin, MA) using a 1/16″ PEEK union with 1/16″ o.d. tubing sheaths used with both the syringe and the PFA tubing (all tubing and connectors from Idex, Oak Harbor, WA). To generate flow, a Chemyx Fusion 200 syringe pump (Stafford, TX) was operated in withdraw mode at a rate of 2.5 µL/min. The droplets were formed by moving the tube inlet between the oil layer and the two color wells in an alternating fashion. The motors were operated at 75 RPM with a 0 s delay within each well. The droplet stream was monitored on a stereo microscope (1-4x magnification) coupled to a Moticam 1080 HD camera (National Optical & Scientific Instruments Inc., Schertz, TX). This video is included (at 2x playback speed) as **Figure S1**. Signal was recorded as RGB intensity over time with ImageJ [33] (National Institutes of Health, Bethesda, MD) and the “stack interleaver” with “ratio profiler” function in the “ImageJ for Microscopy” plug-in [34] (McMaster Biophotonics Facility, Hamilton, ON). Data was analyzed and plotted using Microsoft Excel (Redmond, WA) and Igor Pro 6.0 (Wavemetrics, Inc., Lake Oswego, OR). Red and green intensity values over time, selected to show individual droplet patterns as the sampler moved between the wells, are shown in Fig. 58. The average combined RSD value calculated for the intensity of 15 red droplets and 15 green droplets generated at an overall rate of approximately 0.5 Hz is 2.23%.

## 8 Conclusions

This report details the design and construction of two mechanisms for movement control that can be used for chemical sampling: 3-axis stepper motors and SCARA-based servomotors. Both models provide adequate precision to move to individual wells with a 96-well plate, although higher precision was observed for the 3-axis design. The advantage of the SCARA design is reliance upon 3D printed parts, as it simplifies the construction compared to the need to modify commercial parts like in the 3-axis sampler. Once the relevant parts are obtained (purchased, 3D printed, and/or cut), both builds can be completed in approximately 3–4 h, and similar skill levels are required to construct each design. Based on their open-source design principles, both systems can be further adapted to suit more specific needs for robotic movement in chemical measurement systems (an increasing need in the field [35], [36], [37], [38]), and can be directly applied for the generation of segmented flow droplet streams from 96-well plates that can be used in HTS applications.

## Declaration of Competing Interest

The authors declare that they have no known competing financial interests or personal relationships that could have appeared to influence the work reported in this paper.

## Footnotes

Appendix A Supplementary data to this article can be found online at https://doi.org/10.1016/j.ohx.2021.e00220.
