# Supplementary material for: Comparison of design approaches for low-cost sampling mechanisms in open-source chemical instrumentation
Source: HardwareX. 2021 Aug 10;10:e00220. doi: 10.1016/j.ohx.2021.e00220 (PMC8452234; doi:10.1016/j.ohx.2021.e00220)
Supplement: Supplementary data 1 [file mmc2.pdf]

Comparison of Design Approaches for Low-Cost Sampling Mechanisms in Open-Source Chemical Instrumentation

Supporting Information

S1. Assembling the Control Board PCB

It is simplest to assemble the Control Board and Power Supply PCBs by starting with the shorter components (the ones that lie closest to the board, like resistors and LEDs) and progressing to the taller components like the headers, jacks and switches. Some users may prefer to solder the 20 pin header to the back side of the board first, since it is challenging to fit a soldering iron around the components on the top of the board once they have been soldered into place.

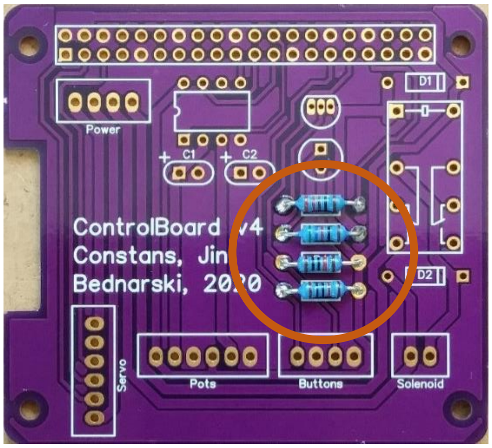

Figure 1: Start by soldering the four resistors onto the Control Board

1. Resistors

Solder the resistors R1 – R4 onto the PCB as shown in Figure 1. Polarity does not matter, but it will make the values easier to read if the tolerance bands are all placed on the right-hand side.

- R1 (221Ω) red / red / brown / black
- R2 (562Ω) green / blue / red / black
- R3, R4 (10k) brown / black / black / red

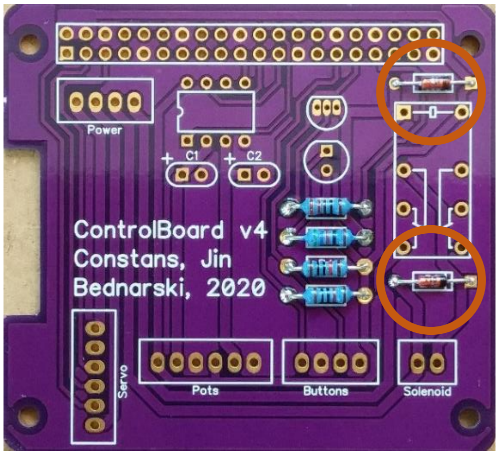

Figure 2: Solder the diodes onto the board, being careful to observe the polarity

2. Diodes

Solder the diodes D1 and D2 onto the board as shown in Figure 2. Be sure to orient the diodes so that the black stripe faces to the right.

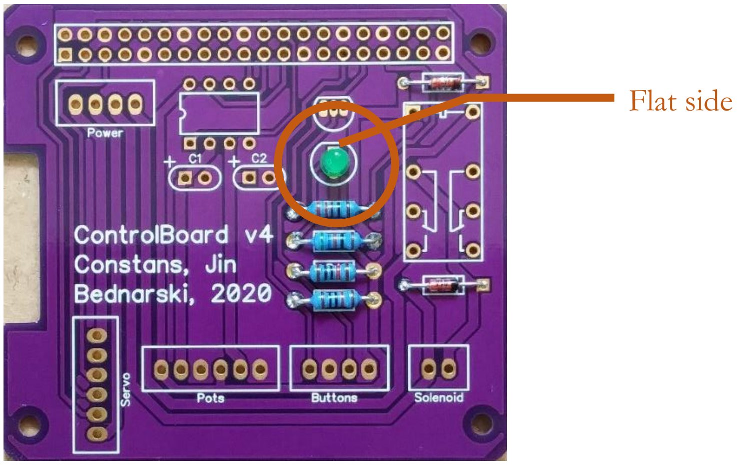

Figure 3: Solder the LED onto the board, keeping the flat side pointing upward

3. LED

Solder the LED, making sure that the flat face is pointing upward as shown in Figure 3.

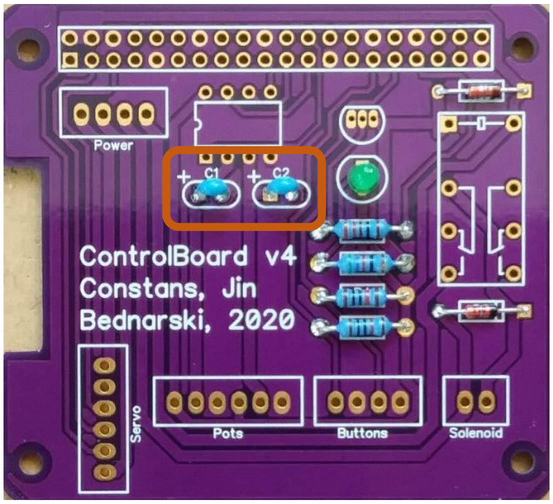

Figure 4: Install the ceramic capacitors, which are not polarized

4. Capacitors

Install C1 and C2 as shown in Figure 4. These ceramic capacitors are not polarized, so their orientation does not matter.

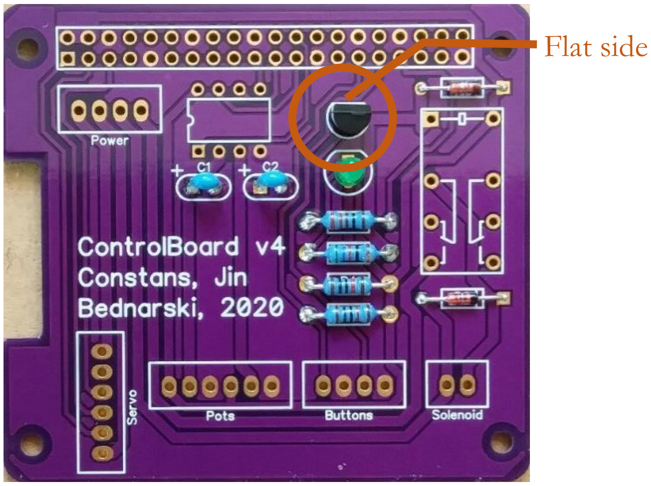

Figure 5: Install the BC546 transistor with the flat side facing up.

5. Transistor

Install the BC546 transistor as shown in Figure 5. Make sure that the flat side is facing upward.

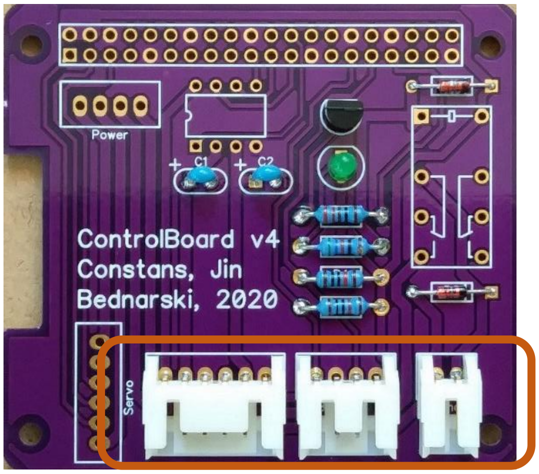

Figure 6: Install the output headers at the bottom of the board.

6. Output headers

Install the three output right-angle headers as shown in Figure 6.

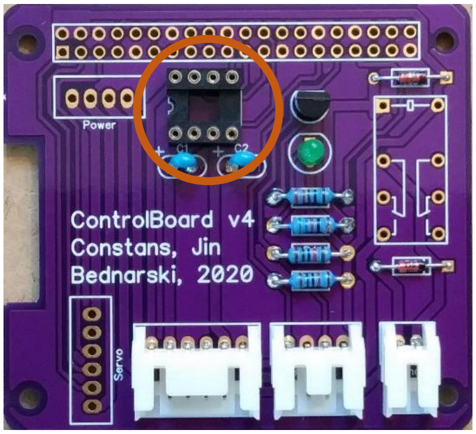

Figure 7: Install the 8-pin socket for the ADC chip, but don't insert the chip into the socket yet.

7. Socket for ADC chip

Install the eight-pin socket for the ADC chip as shown in Figure 7. Make sure that the notch in the socket is on the right side. Do not plug the ADC chip into the socket until all soldering is complete.

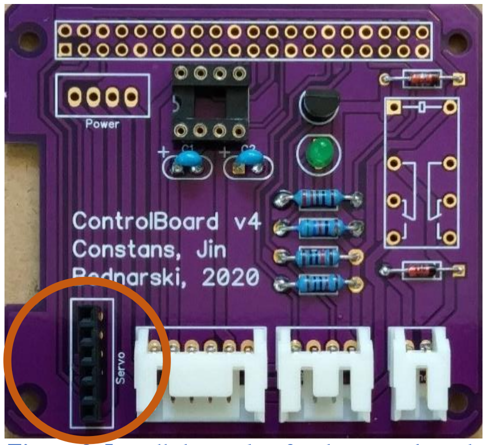

Figure 8: Install the socket for the servo board.

8. Servo socket
Install the socket for the servo control board as shown in Figure 8. The servo control board will lie across the output headers.

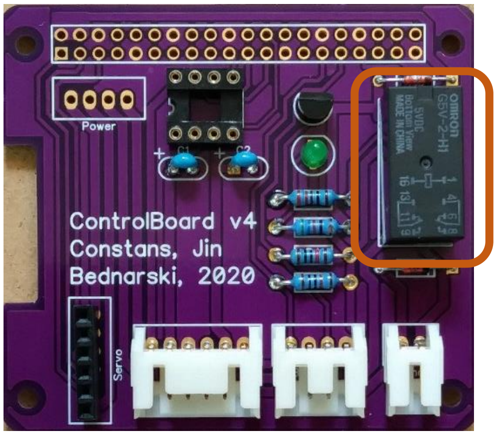

Figure 9: Install the relay for the solenoid.

9. Relay
Install the solenoid relay as shown in Figure 9.

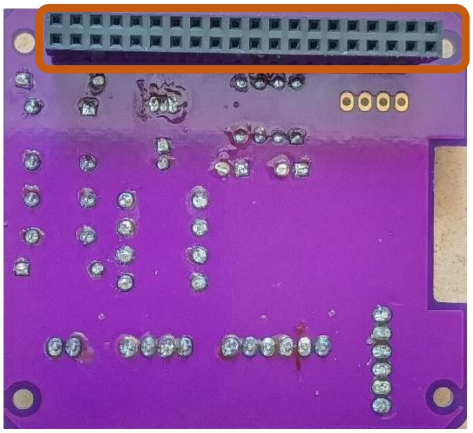
Figure 10: Install the Raspberry Pi header on the back side of the board.

10. 20-pin header for Raspberry Pi

Flip the board over and solder the 20-pin header onto the back side as shown in Figure 10. The female side of the header must be facing downward.

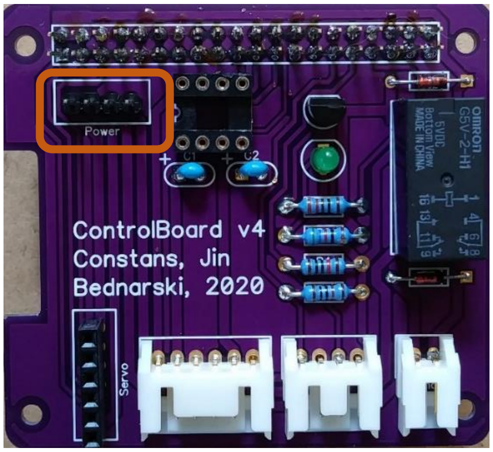

Figure 11: Install the power supply header on the top side of the board.

11. Power supply header

Flip the board back over and solder the power supply header as shown in Figure 11. This will plug into the socket on the lower side of the power supply board.

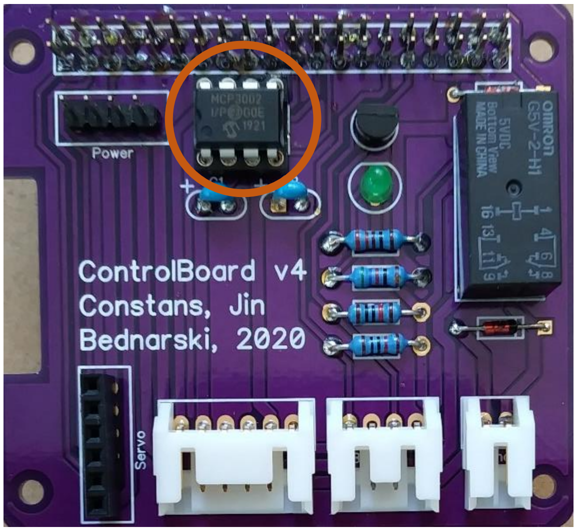

Figure 12: Plug the ADC chip into the socket once all soldering is complete.

12. Analog to Digital Converter

Finally, plug the MCP3002 ADC chip into its socket as shown in Figure 12. The Control Board is now complete.

S2. Assembling the Power Supply Board

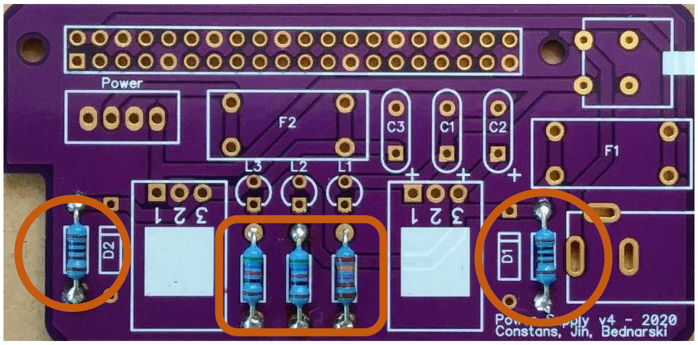

Figure 13: Solder the resistors onto the board.

13. Resistors

Install resistors R1 – R5 as shown in Figure 13. The color codes are as follows:

- R1, R2 562Ω Green / Blue / Red / Black
- R3, R4 1k Brown / Black / Black / Brown
- R5 2.37k Red / Orange / Violet / Brown

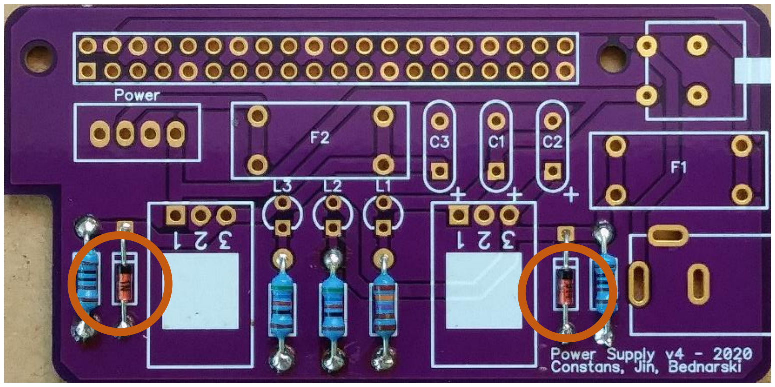

Figure 14: Solder the diodes onto the board, paying careful attention to their polarity.

14. Diodes

Install the 1N4149 diodes as shown in Figure 14. The black stripe must be facing upwards.

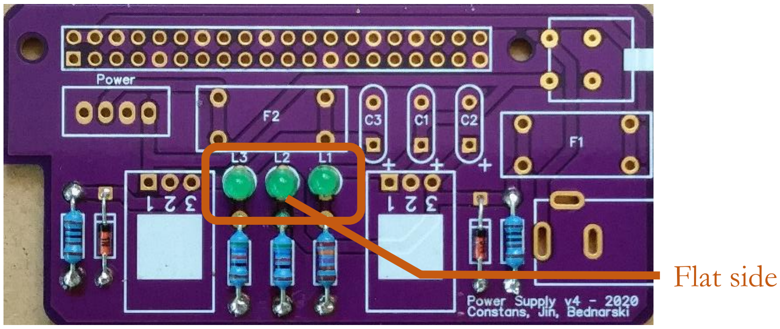

Figure 15: Install the three LEDs on the board, with the flat side facing downward.

15. LEDs
Install the three LEDs with the flat sides facing downward as shown in Figure 15.

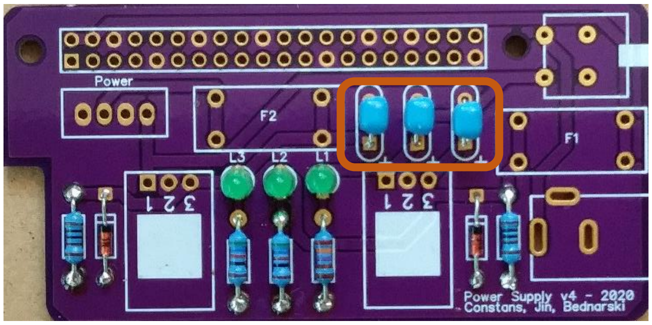

Figure 16: Install the three ceramic capacitors, which are nonpolarized.

16. Capacitors
Install the three 22µF ceramic capacitors as shown in Figure 16. These capacitors are not polarized, so the orientation does not matter.

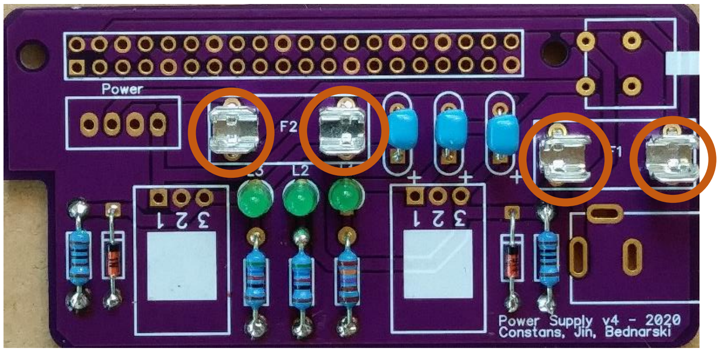

Figure 17: Install the fuse holders, ensuring that the small tabs are facing outward.

17. Fuseholders
Install the four fuseholders as shown in Figure 17, paying particular attention to orientation (the metal tabs should be facing outwards on each fuseholder.

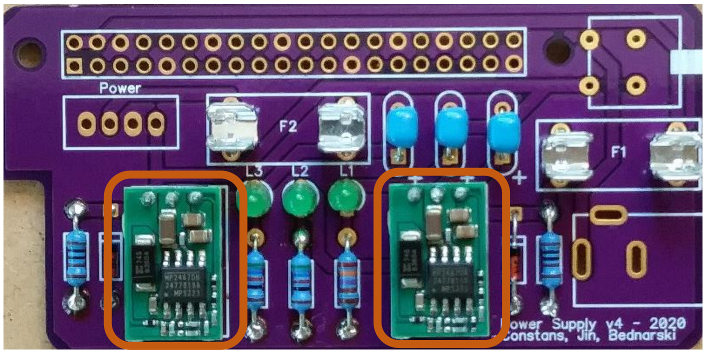

Figure 18: Install the voltage regulators.

18. Voltage Regulators

Install the two OKI-78SR-5 voltage regulators as shown in Figure 18.

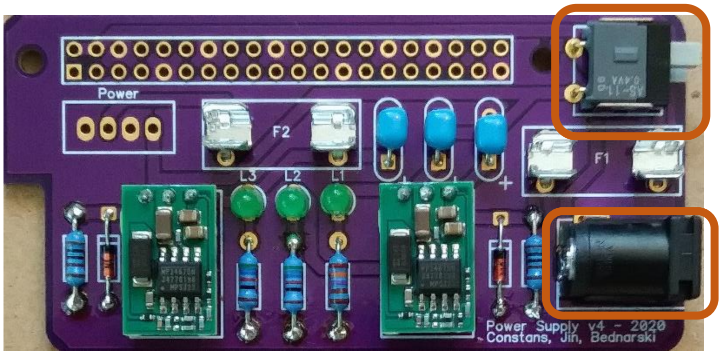

Figure 19: Install the power switch and power jack.

19. Switch and Power Jack

Install the on/off switch and power jack as shown in Figure 19.

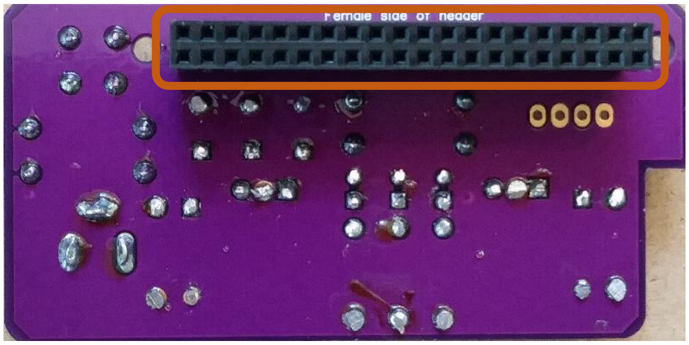
Figure 20: Install the Raspberry Pi header on the back side of the board.

19. 20-pin Header for Raspberry Pi

Flip the board over and install the 20-pin header for the Raspberry Pi as shown in Figure 20. The female side of the header should be pointing downward.

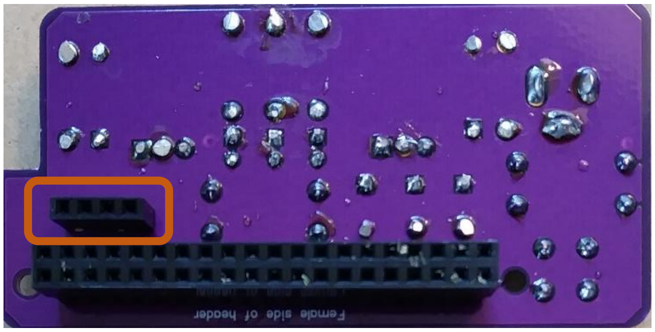

Figure 21: The power supply socket should also be on the back side of the board.

21. Power supply socket

With the board still flipped over, install the power supply socket as shown in Figure 21. The female side of the socket should be pointing downward. The soldering of the power supply board is now complete.
